# Infant airway microbiota and topical immune perturbations in the origins of childhood asthma

**DOI:** 10.1038/s41467-019-12989-7

**Published:** 2019-11-01

**Authors:** Jonathan Thorsen, Morten A. Rasmussen, Johannes Waage, Martin Mortensen, Asker Brejnrod, Klaus Bønnelykke, Bo L. Chawes, Susanne Brix, Søren J. Sørensen, Jakob Stokholm, Hans Bisgaard

**Affiliations:** 1 COPSAC, Copenhagen Prospective Studies on Asthma in Childhood, Herlev and Gentofte Hospital, University of Copenhagen, Copenhagen, Denmark; 2 Section of Chemometrics and Analytical Technology, Department of Food Science, University of Copenhagen, Copenhagen, Denmark; 3 Section of Microbiology, Department of Biology, University of Copenhagen, Copenhagen, Denmark; 4 Disease Systems Immunology, Department of Biotechnology and Biomedicine, Technical University of Denmark, Lyngby, Denmark; 5 Department of Pediatrics, Naestved Hospital, Naestved, Denmark

**Keywords:** Microbiome, Asthma, Translational research

## Abstract

Asthma is believed to arise through early life aberrant immune development in response to environmental exposures that may influence the airway microbiota. Here, we examine the airway microbiota during the first three months of life by 16S rRNA gene amplicon sequencing in the population-based Copenhagen Prospective Studies on Asthma in Childhood 2010 (COPSAC_2010_) cohort consisting of 700 children monitored for the development of asthma since birth. Microbial diversity and the relative abundances of *Veillonella* and *Prevotella* in the airways at age one month are associated with asthma by age 6 years, both individually and with additional taxa in a multivariable model. Higher relative abundance of these bacteria is furthermore associated with an airway immune profile dominated by reduced TNF-α and IL-1β and increased CCL2 and CCL17, which itself is an independent predictor for asthma. These findings suggest a mechanism of microbiota-immune interactions in early infancy that predisposes to childhood asthma.

## Abstract (web summary)

Here, Thorsen et al. examine the microbiota during the first three months of life in a cohort of 700 children and find that microbial diversity and the relative abundances of *Veillonella* and *Prevotella* in the airways at one month of age are associated with topical immune mediators and asthma by age 6 years.

## Introduction

Asthma has risen to epidemic proportions during the past half-century^1–3^. Like many other chronic inflammatory non-communicable diseases (NCDs), it is believed to originate in early life when the child’s immune system is developing^4–6^. Unfavorable or insufficient microbial stimulation of the developing immune system has been suggested to increase the propensity for chronic inflammation involved in development of asthma and other NCDs^7–11^. Epidemiological studies have found associations between asthma risk and several environmental exposures related to the microbiota. Living with farm animals, older children in the home and daycare attendance have all been associated with a decreased risk^12,13^, whereas delivery by cesarean section may increase the risk^14,15^.

All body compartments harbor their own distinct compositions of microbes that affect local immune cells^16^. This has driven a search for microbial signatures related to human health and disease^17–20^. The airways contain a plethora of microbes, representing a diverse biogeographical continuum through the respiratory tract^21–24^. We have previously shown associations between neonatal pathogenic bacterial airway colonization and later asthma^25^, and described the airway immune profiles in neonates colonized with such bacteria^26^. However, these studies were limited by the available culturing technique, which does not capture the complexity of the entire airway microbiota.

Here, we therefore examine the early-life airway microbiota using 16S ribosomal RNA (rRNA) gene amplicon sequencing and analyze its relation to the development of asthma by age 6 years among children participating in the unselected Copenhagen Prospective Studies on Asthma in Childhood 2010 (COPSAC_2010_) birth cohort. Simultaneously, we analyze the topical immune profile from airway mucosal lining fluid hypothesizing that specific bacterial taxa may increase the risk of asthma through perturbations of the developing airway immune system. We show that the diversity and composition of the airway microbiota, and the relative abundances of *Veillonella* and *Prevotella*, at age 1 month are associated with the development of asthma by age 6 years. Furthermore, these and other taxa together contribute to the risk of asthma in a multivariable sparse partial least squares (sPLS) model. From this model, we derive a bacterial asthma score for each child based on abundances of these specific taxa, which is associated with a specific topical immune profile characterized by reduced tumor necrosis factor-α (TNF-α) and interleukin-1β (IL-1β) and increased Chemokine (C-C motif) ligand 2 (CCL2) and CCL17. Finally, this immune profile exhibits both a shared and a unique, independent contribution to the risk of asthma. Collectively, these findings indicate that the early-life airway microbiota may predispose to the development of asthma later in childhood through dynamic interactions with the developing immune system.

## Results

### Sample characteristics

We successfully sampled and sequenced the V4 region of the 16S bacterial rRNA gene in 1788 airway aspirates (≥2000 reads) from 544 (77.7%) of the 700 children in the COPSAC_2010_ cohort at age 1 week, 622 (88.9%) at age 1 month, and 622 (88.9%) at age 3 months. In total, 695 children (99.3%) participated with at least one sample. The children were prospectively monitored for asthma development from birth with frequent clinical visits. Full clinical follow-up during the first 6 years of life was available for 644 children (92.0%) and 146 children (22.7%) developed asthma in this period.

### Airway microbial composition in early life

The airway samples had a median sequencing depth of 45,334 (interquartile range (IQR) [22,216–70,667]) reads. We identified 3,582 unique operational taxonomic units (OTUs) from 574 genera, with a median richness of 70 OTUs [53–91] per sample. Alpha diversity increased from 1 week (median Shannon index 1.18, IQR [0.67–1.64]) to 1 month (1.40 [0.93–1.82]) and 3 months (1.58 [1.13–2.04]), *p* < 0.0001. The airway microbial populations were dominated by taxa from the phyla Firmicutes and Proteobacteria at all time-points, specifically the genera *Staphylococcus*, *Streptococcus*, *Moraxella*, *Haemophilus*, and *Corynebacterium* (Supplementary Fig. 1). These genera showed profound changes over time, most noticeably a decrease in *Staphylococcus*, and increases in *Streptococcus*, *Moraxella*, and *Haemophilus*. Less abundant genera also varied substantially over time, in particular *Neisseria*, *Rothia*, *Fusobacterium*, and *Prevotella*, which all increased several fold from the 1-week to the 3-month samples. These changes point to a rapid and dynamic early-life development of the airway microbiota.

### Microbial diversity, differential abundances, and asthma

**Fig. 1:**
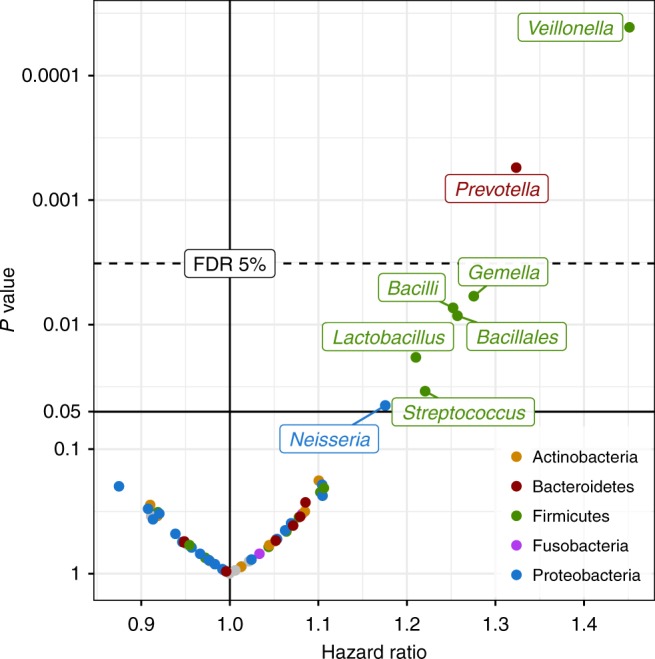
Differential abundance and asthma. Hazard ratios and corresponding *p* values from Cox proportional hazards regression models using log-transformed relative abundances for each genus as a predictor for asthma by age 6 years. Dashed line indicates 5% false discovery rate (FDR) cutoff. Colored by taxonomic phylum. *n* = 573

We then compared the airway microbiota at the three early-life time-points to asthma development in the first 6 years of life. At age 1 month, both an increased alpha diversity and a difference in beta diversity were found in children who developed asthma in the first 6 years of life compared to those who did not (Table 1 and Supplementary Fig. 2). Higher relative abundances of the bacterial genera *Veillonella* (Cox regression, hazard ratio (HR) 1.45 per standard deviation (SD), 95% confidence interval [1.21–1.73], *p* < 0.0001, false discovery rate (FDR) *q* value = 0.003, *n* = 573) and *Prevotella* (HR 1.32 [1.13–1.55], *p* = 0.0005, *q* = 0.017, *n* = 573) were associated with asthma development (Fig. 1 and Supplementary Table 1). At ages 1 week and 3 months, there were no significant associations between alpha or beta diversity or any specific taxa and development of asthma after FDR adjustment (Supplementary Table 2).

**Table 1:** Alpha and beta diversity and asthma

(A) Alpha diversity			
Metric	Asthmatics (median [IQR])	Non-asthmatics (median [IQR])	*p* Value
Shannon index	1.56 [1.11–1.90]	1.38 [0.87–1.80]	0.0046
Richness at 2000 reads	30 [25–36]	27 [21–34]	0.00044
Richness at 10,000 reads	49 [40–57]	43 [34–54]	0.0017

(B) Beta diversity			
Metric	*F* statistic	*p* Value	
Bray–Curtis	2.21	0.016	
UniFrac, weighted	2.27	0.046	

Diversity estimates from 1-month airway samples between children with asthma in the first 6 years of life, and children without asthma. *n* = 573. (A) Alpha (within-sample) diversity estimates. (B) Beta (between-sample) diversity estimates. *F* statistic is calculated as between-group variance vs. within-group variance

### Multivariate bacterial asthma score

**Fig. 2:**
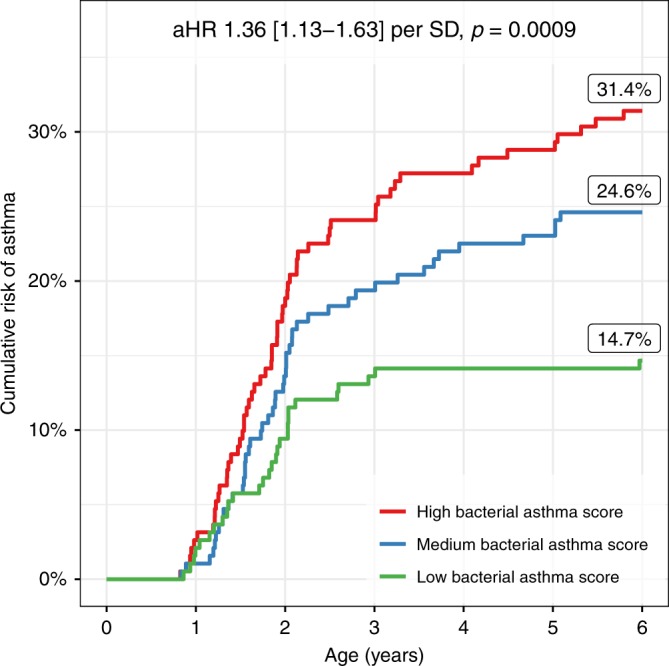
Bacterial asthma score and asthma. Sparse partial least-squares (sPLS) model between genus-level relative abundances at 1 month of age and asthma by age 6 years. Kaplan–Meier curve showing cumulative risk of asthma by bacterial asthma score, divided into tertiles. *n* = 573 (191 in each tertile group). Adjusted hazard ratio (aHR) and 95% confidence interval corresponds to each standard deviation (SD) of the continuous score from the sPLS model, adjusted for paternal asthma, season of birth, and siblings in a Cox regression (*n* = 554). The displayed percentages are the Kaplan–Meier estimates of asthma risk at 6 years in each tertile group

At 1 month of age, we found several groups of microbial genera with moderate to high pairwise correlations (Supplementary Fig. 3). To further examine associations with asthma, we therefore constructed a cross-validated sparse PLS model to identify jointly relevant taxa at age 1 month predicting asthma by 6 years of age, which resulted in a one-component model based on relative abundances of seven genera (Supplementary Fig. 4). These were *Veillonella* (relative importance 28.1%), *Prevotella* (23.7%), *Gemella* (16.3%), *Bacillales* incertae sedis (12.3%), *Bacilli* incertae sedis (12.2%), *Streptococcus* (5.4%), and *Lactobacillus* (2.1%) (Supplementary Table 3). We defined a continuous bacterial asthma score based on the model (Supplementary Fig. 5), which was associated with paternal asthma (higher score), season of birth (higher score in winter), and having older siblings (lower score), but not with any other environmental or heritable factors (Supplementary Table 4). We therefore adjusted the asthma association models for paternal asthma, older siblings, and season of birth. The bacterial asthma score was associated with asthma development by age 6 years (Cox regression, adjusted HR per SD 1.36 [1.13–1.63], *p* = 0.0009, *n* = 573, Fig. 2). The bacterial asthma score was associated with both transient early, persistent, late-onset phenotypes and current asthma at age 6 years (Table 2), with a tendency of higher effect estimates in the late-onset and current at age 6 groups. When stratifying for allergic sensitization to common inhalant allergens at age 6 years, we found a significant association in both strata, and a tendency of higher effect estimates in the sensitized vs. non-sensitized group (Table 2). However, this difference was not significant in an interaction test (*p* = 0.36). The bacterial asthma score was not associated with allergic sensitization at 6 years (logistic regression, odds ratio (OR) 0.94 [0.75–1.17], *p* = 0.57), nor when restricting the analysis to children without asthma in the first 6 years of life (OR 0.88 [0.675–1.13], *p* = 0.31).

**Table 2:** Microbiota–asthma association according to age at onset, persistence, and sensitization status

Phenotype	Definition	OR	aOR	95% CI	*p* Value	*N* cases/controls
Asthma ever	Asthma by age 6	1.50	1.44	1.17–1.79	0.00062	135/438
Current asthma at 6 years	Active diagnosis at age 6	1.67	1.61	1.15–2.30	0.0072	44/438
Transient early	Diagnosis before age 3, remission before age 6	1.37	1.33	1.04–1.72	0.025	81/438
Persistent	Diagnosis before age 3, still ongoing at age 6	1.45	1.44	0.95–2.20	0.091	28/438
Late onset	Diagnosis after age 3	2.10	1.92	1.23–3.11	0.0056	26/438

Phenotype	Definition	HR	aHR	95% CI	*p* Value	*N* cases/controls
Asthma; sensitized children	Stratified by positive SPT or sIgE at age 6	1.64	1.67	1.03–2.69	0.037	25/74
Asthma; non-sensitized children	Stratified by negative SPT or sIgE at age 6	1.30	1.27	1.00–1.61	0.053	73/262
Asthma; sensitization not assessed	Missing sensitization data	1.53	1.47	0.99–2.21	0.059	37/102

*OR* odds ratio, *CI* confidence interval, *HR* hazard ratio, *SPT* skin prick test, *sIgE* specific immunoglobulin E

Estimates from logistic regression (OR, CI) or Cox proportional hazards regression (HR) between the bacterial asthma score and asthma, according to age-dependent phenotype and allergic sensitization status to inhalant allergens (SPT, sIgE). Crude and adjusted (aOR/aHR) estimates provided, after adjustment for season of birth, paternal asthma, and siblings in the home. Confidence intervals and *p* values pertain to adjusted analyses. We found no evidence of an interaction effect between the bacterial asthma score and allergic sensitization (*p* = 0.36)

### Bacterial asthma score and topical airway immune profiles

**Fig. 3:**
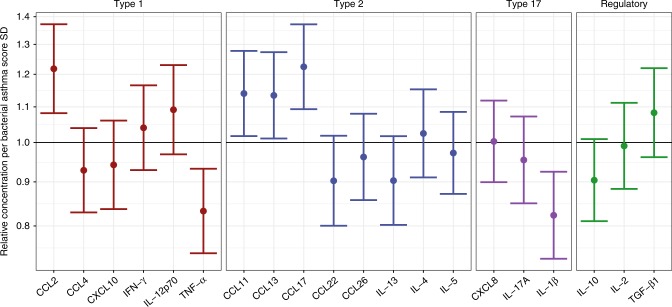
Airway immune profile and bacterial asthma score. Associations between the bacterial asthma score, based on Veillonella, Prevotella, Gemella, Bacilli incertae sedis, Bacillales incertae sedis, Streptococcus, and Lactobacillus, and upper airway mucosal immune mediators. Linear models show that the bacterial asthma score is associated with several immune mediators, expressed as relative concentration ratios of immune mediators per standard deviation (SD) increase in bacterial asthma score, *n* = 499. Error bars indicate 95% confidence intervals. Associations are adjusted for collinearity with other bacteria

We analyzed the relationship between the bacterial asthma score and topical immune mediators from the airway mucosal lining fluid, sampled concurrently at 1 month of age in 499 (80.2%) of the 622 children with available microbial samples (Supplementary Fig. 6). We observed positive associations with the chemoattractants CCL17 and CCL2 and negative associations with the cytokines TNF-α and IL-1β (Fig. 3 and Supplementary Table 5). We adjusted the model for common variation in other bacteria to remove the immune effects not directly related to the bacterial asthma score (Supplementary Fig. 7). The associations were further examined in a multivariate sparse PLS model with cross-validation, which showed a strong association between the bacterial asthma score and the immune mediators (Spearman’s correlation 0.18, linear model *p* < 0.0001), with TNF-α, IL-1β, CCL2, and CCL17 being the most important variables in the model (Supplementary Fig. 8). The immune mediator score from this model was independently associated with asthma by age 6 years, even after adjusting for the bacterial asthma score in a mediation analysis that revealed a low degree of mutual mediation (Cox regression, restricted to children with immune mediator data available, *n* = 499; bacterial asthma score alone: HR 1.42 [1.17–1.71], *p* = 0.0003; immune mediator score alone 1.34 [1.11–1.61], *p* = 0.0019; combined analysis, bacterial asthma score 1.37 [1.13–1.67], *p* = 0.0012, immune mediator score aHR 1.29 [1.07–1.55], *p* = 0.008, proportion mediated 10.5% [2.2–28.2%], *p* = 0.0078, see details in Supplementary Table 6).

## Discussion

We found that the diversity and the composition of the airway microbiota in asymptomatic 1-month-old infants were associated with the risk of developing asthma within the first 6 years of life. Specific individual bacterial taxa and a composition enriched with these taxa, in particular *Veillonella*, *Prevotella*, and *Gemella*, increased the risk of developing asthma. These asthma-related bacteria were associated with a distinct topical airway immune profile, which was also associated, independently, with risk of asthma by age 6 years. This suggests a potential involvement of topical immune perturbation linking the early-life airway microbiota to later asthma development.

A major strength of the study was the extensive and prospective clinical assessment of asthma and environmental exposures in the COPSAC_2010_ population-based mother–child cohort^6,27^. Samples were collected in infancy before onset of symptoms and all children were uniformly monitored for development of asthmatic symptoms at a single-center research unit. Furthermore, the diagnostic algorithm and treatment regimen were pre-defined and consistently applied by study pediatricians avoiding the heterogeneity in diagnostics and treatment of preschool asthma that is common in the health care community.

Compared to studies analyzing samples from the nasopharynx^28,29^ or sputum, our technique allows for collection of biomaterial close to the lungs without the need for bronchoscopy or other invasive techniques. This involves sampling of microbes from the topical hypopharyngeal airway mucosa as well as from lower airway secretions transported via mucociliary clearance. Additionally, it is a major strength that we simultaneously investigated the airways’ topical immune signature at 1 month, providing insight into the interaction between the bacterial taxa and the topical immunity. While anatomically distinct and each possessing unique microbiotas, the nose and hypopharynx are close enough to allow a significant exchange of microbes within an individual^22,24,30^. Similar to how circadian fluctuations in the lung microbiota of adults depending on sleeping positions have been hypothesized, 1-month-old infants are often in a horizontal position and it is recommended that they sleep supine, which may facilitate microbial transfer between upper and lower airways and microaspiration^24,31^.

We applied 16S rRNA gene amplicon sequencing to deeply characterize the taxonomic composition of the airway microbiota, which greatly increases the power to detect non-cultivable and/or difficult to culture anaerobic taxa, although at the expense of discrimination at species level. All 16S rRNA regions, including the V4 region used in this study, bias the observed composition due to varying primer affinity and the inter-taxonomic variability of the region in question. Airway samples furthermore contain much lower absolute amounts of bacteria than, for example, stool samples, which may result in an incomplete picture of the resident microbiota. Although these biases may skew the compositional accuracy of the microbiota, they will not interfere with the associations with asthma. The 16S sequencing technique does not allow for direct estimation of microbial genes and their metabolic functional potential, which may underpin some of the associations reported here. Future studies utilizing more detailed meta’omic profiling may provide further mechanistic insight into these associations^32^.

Supervised analyses such as PLS models are designed to find all possible associations in the data, but our application of a cross-validation scheme ensures robust results without overfitting. The inclusion of a constraint to yield sparse results further strengthens this approach to restrict solutions to a limited set of predictors, which are jointly associated with the outcome. The PLS methodology has been developed to account well for highly collinear feature matrices, which makes it well suited to apply to both the microbiota and immune mediator data^33^.

The airway microbiota composition in healthy 1-month-old infants, in particular the relative abundances of the taxa *Veillonella* and *Prevotella*, was associated with later asthma, suggesting a role of such a microbiota composition in asthma pathogenesis. This asthma-associated composition furthermore correlated with lower levels of topical pro-inflammatory airway immune mediators (IL-1β and TNF-α), which may characterize an inefficient anti-bacterial response, and higher levels of monocyte and T-cell recruiting chemoattractants (CCL2 and CCL17). This could reflect a delayed general induction of immune stimulation exerted by the microbiota, which has been suggested to predispose to long-term immune dysregulation, low-grade inflammation, and immune-mediated NCDs later in life^5,8,34^. Notably, although we assayed mucosal lining fluid from the nose, the observed associations may reflect systemic mucosal immune response patterns. The airway immune profile identified via the bacterial asthma score was independently associated with asthma, suggesting this as a general risk factor for asthma development potentially affected by other environmental exposures and/or genetic factors. The association with an immune pattern suggests that these taxa, despite being rare compared to other taxa, may exert clinically relevant effects on the host through sensitive immune responses.

Specific functions of *Veillonella* and *Prevotella* may have direct implications for the current findings. They are both Gram-negative and largely anaerobic bacterial taxa prevalent in the gut, and also key members of the oral microbiota^16^ from where they presumably migrate to the lower airways through microaspiration^35^. Notably, they have both been identified in high abundances in the lungs of subjects with chronic obstructive pulmonary disease^36^ and associated with subclinical airway inflammation in healthy adults^35,37^. Both *Veillonella* and *Prevotella* harbor the less immune-stimulatory penta-acylated lipopolysaccharide^38^ that limits their capacity for activation of immune cells^8,39^. This could lead to an inappropriate topical host immune response, which is supported by our finding of reduced secretion of airway TNF-α and IL-1β in children with high levels of these asthma-associated bacteria, and increased levels of CCL2 and CCL17, of which the latter has previously been associated with childhood asthma^40^. Such interpretation favors the notion that the observed airway microbiota composition leads to perturbations of the topical immune response and not vice versa.

A recent study of the nasopharyngeal microbiota in 234 children from the Australian Childhood Asthma Study cohort aged 2–12 months found that the microbial composition characterized by 16S sequencing was dominated by *Haemophilus*, *Streptococcus*, *Moraxella*, *Alloiococcus*, *Corynebacterium*, and *Staphylococcus*^28^. Of these, *Streptococcus* was associated with a questionnaire-based chronic wheeze phenotype at 5 years. Lesser taxa, such as *Veillonella* and *Prevotella*, were identified in the samples, but not tested against disease. The larger ecosystem of the airway microbiota may exhibit similar associations across different parts of the respiratory tract due to inter-site communication, consistent with previous reports^22,26^.

In our older COPSAC_2000_ cohort, we reported associations between airway colonization with *Moraxella catarrhalis*, *Haemophilus influenzae*, and *Streptococcus pneumoniae* at age 1 month and later asthma^25^. In the present study, *Streptococcus* was associated with later asthma and contributed to the bacterial asthma score, whereas *Moraxella* and *Haemophilus* were not. This is probably due to different methodologies: 16S rRNA gene amplicon sequencing vs. culturing, where the first approach results in reduced detection specificity at the species level, but increased sensitivity to non-cultivable genera. Additionally, the COPSAC_2000_ cohort is a high-risk cohort born to mothers with asthma. Finally, it may be that the members of the airway microbiota that carry the association with asthma are not specific, but simply reflect latent community structures.

Both the airway microbiota and the infant immune system undergo dramatic development from birth^21,41,42^. Bearing this in mind, any consequences of the airway microbiota for the developing immune system may be contingent upon a specific age window, yielding different biological effects at different ages. The absence of any associations with later asthma from the 1-week and 3-month microbiota supports a hypothesis of a lasting immune imprinting exerted in a narrow open window of development in early life around 1 month of age^25^. We found lower bacterial asthma scores at age 1 month in children with older siblings and higher scores in children born in winter. We therefore speculate that a part of the known sibling^13^ and seasonal effects^43^ on asthma risk could be due to the airway microbiota acting in the same narrow age frame.

We found that the association between the airway microbiota and asthma was present regardless of age at onset or sensitization at 6 years, with a non-significant tendency of higher effect estimates for late-onset and sensitized phenotypes. Whether these associations will stretch further into school age, adolescence, and even adulthood is unclear, and further follow-up of the cohort is therefore highly warranted.

The observational design of the study precludes direct evaluation of directionality or causality. It remains elusive whether the early airway bacterial composition may contribute to asthma pathogenesis through topical immune perturbations, whether the airway immune composition predisposes to asthma through inappropriate responses to airway bacteria and other environmental exposures, or a latent host phenotype, unmeasured confounder, or complex unresolved immune-microbiota interplay is the true cause. Furthermore, any bacteria-related effect may be contingent upon a susceptible host genetic architecture. If, however, the bacteria are later determined to be the true causal agents in these associations, our data suggest a potential for asthma prevention in early life by targeted manipulation of the developing airway microbiota.

We demonstrated an association between the composition of the airway microbiota in asymptomatic 1-month-old infants and the risk of developing asthma by age 6 years. Simultaneously, we observed that this composition associated with a specific airway immune profile, which was independently associated with asthma. These findings support the hypothesis that specific microbial taxa, in part through early-life interactions with the host immune system, may predispose to asthma later in childhood.

## Methods

### Study population and sample collection

The study was embedded in the population-based COPSAC_2010_ prospective mother–child cohort of 700 children and their families followed from week 24 of pregnancy^6,27,44^. Hypopharyngeal aspirates were obtained at ages 1 week, 1 month, and 3 months, using a soft suction catheter passed through the nose. The children attended scheduled visits to the COPSAC clinic, where they were assessed by study pediatricians at ages 1 week, 1, 3, and 6 months, and 1, 1.5, 2, 2.5, 3, 4, 5, and 6 years. Acute care visits were arranged whenever the children experienced lung or skin symptoms and the research clinic was de facto acting as family practitioner for the children.

### Ethics

We are aware of and comply with recognized codes of good research practice, including the Danish Code of Conduct for Research Integrity. We comply with national and international rules on the safety and rights of patients and healthy subjects, including Good Clinical Practice (GCP) as defined in the EU’s Directive on GCP, the International Conference on Harmonization’s (ICH) GCP guidelines and the Declaration of Helsinki. We follow national and international rules on the processing of personal data, including the Danish Act on Processing of Personal Data and the practice of the Danish Data Inspectorate. The study was conducted in accordance with the guiding principles of the Declaration of Helsinki and was approved by the Capital Region of Denmark Local Ethics Committee (H-B-2008-093), and the Danish Data Protection Agency (2015-41-3696). Both parents gave oral and written informed consent before enrollment.

### Clinical endpoints and covariates

Asthma was diagnosed using a pre-defined validated quantitative symptom algorithm^27,45^, based on parental registration of troublesome lung symptoms on structured daily diary cards from birth, verified by study pediatricians at each clinic visit. This ensured that symptoms were compatible with asthma, including exercise-induced symptoms, prolonged nocturnal cough, and coughing outside common cold. Children with a symptom burden of at least five episodes within 6 months each lasting at least 3 days, 4 weeks of continuous symptoms, or a severe exacerbation requiring hospitalization or oral corticosteroid treatment were prescribed a 3-month trial of inhaled corticosteroid (ICS). Asthma was diagnosed in children with relapse of symptoms upon cessation of the ICS trial. Remission was defined by the absence of relapse after 12 months since last ICS treatment. For the present study, we further defined four subgroups of asthmatics: a transient early, a persistent, and a late-onset asthma phenotype^46^, as well as current asthma at age 6 years.

Information on hereditary and environmental factors, including parental history of asthma and allergy, antibiotics consumption, delivery mode, pets, smoking, older siblings, and breastfeeding were obtained by parental interviews at the scheduled visits.

Allergic sensitization was assessed by skin prick test (SPT) to common inhalant allergens (*Alternaria* spp., birch, cat, *Cladosporium* spp., *D. farinae*, *D. pteronyssinus*, dog, grass, horse and mugwort) and specific immunoglobulin E (sIgE) ≥0.35 kUA/l in blood samples to common inhalant allergens (*D. pteronyssinus* (d1), cat (e1), horse (e3), dog (e5), grass (g6), birch (t3), mugwort (w6), *Cladosporium herbarum* (m2), *Aspergillus fumigatus* (m3), *Alternaria tenuis* (m6)). Both were assessed at 6 years of age. Allergic sensitization to inhalant allergens was defined as one or more positive tests in either SPT or sIgE.

The mothers were enrolled in two pregnancy factorial-designed randomized controlled trials of fish oil and vitamin D3^6,44^, a subgroup were enrolled in a randomized comparison of influenza A/California/2009 vaccines^47,48^, and children with persistent wheeze were randomized to azithromycin or placebo during acute exacerbations^49^.

### Sequencing and bioinformatics

We examined the airway microbiota using a 16S rRNA gene amplicon sequencing protocol targeting the V4 region^21^: the aspirates were diluted in 1 ml sterile 0.9% NaCl and transported to the microbiological laboratory at Statens Serum Institut (Copenhagen, Denmark), where they were separated into 150 µl aliquots and stored at −80 °C until DNA extraction. DNA was extracted from 1 × 150 µl aliquots per sample using the PowerMag^®^ Soil DNA Isolation Kit optimized for epMotion^®^ (MO-BIO Laboratories Inc., Carlsberg, CA, USA) using the epMotion^®^ robotic platform model (Eppendorf, Hamburg, Germany) under the manufacturer’s protocol. All measurements were taken from distinct samples. At least one DNA extraction negative control was included in each 96-well plate, by adding 150 μl of molecular grade water (Sigma-Aldrich, Merck, Germany) instead of a sample. DNA concentrations were determined using the Quant-iTTM PicoGreen^®^ quantification system (Life Technologies, CA, USA). Extracted DNA was stored at −20 °C.

16S rRNA gene amplification of the hypervariable V4 region was performed over two PCR steps. First, amplification of the 16S rRNA gene, using broad range primers (515F (5′-GTGCCAGCMGCCGCGGTAA-3′) and 806R (5′-GGACTACHVGGGTWTCTAAT-3′)), with a reaction mixture consisting of 1× AccuPrime PCR Buffer II, 0.6 U AccuPrime *Taq* DNA Polymerase (Invitrogen, Life technologies, CA, USA), 0.5 µM primer 515F, 0.5 µM primer 806R, 2.0 µl template DNA, and molecular grade water (Sigma-Aldrich, Merck, Germany) to a total volume of 20.0 µl per sample. Reactions were run in a 2720 thermal cycler (Applied Biosystems^®^, Life Technologies, CA, USA) according to the following cycling program: 2 min of denaturation at 94 °C, followed by 30 cycles of 20 s at 94 °C (denaturing), 30 s at 56 °C (annealing), and 40 s at 68 °C (elongation), with a final extension at 68 °C for 5 min. For each plate, a negative template-free control and a positive control containing 2.0 µl DNA from a known bacterial mock community (1.0 ng/µl; HM-782D, BEI Resources, VA, USA) were included. The PCR products were quantified using the Quant-iT™ PicoGreen^®^ quantification system (Life Technologies, CA, USA) and samples with a PCR product concentration above 6.0 ng/µl were diluted to ~3.0–6.0 ng/µl prior to further analysis. Sequencing primers and adaptors were added to the amplicon products in the second PCR step: 2.0 µl of the diluted amplicons were mixed with a reaction solution consisting of 1× AccuPrime PCR Buffer II, 0.6 U AccuPrime *Taq* DNA Polymerase (Invitrogen, Life Technologies, CA, USA), 0.5 µM fusion forward and 0.5 µM fusion reverse primer, and molecular grade water (Sigma-Aldrich, Merck, Germany) (total volume 20 µl). The PCR was run according to the cycling program above, except with a reduced cycling number of 15.

The amplification products were purified with Agencourt AMPure XP Beads (Beckman Coulter Genomics, MA, USA) using 0.7× volume beads and quantified as described above. Equimolar amounts of the amplification products were pooled together in a single tube. The pooled DNA samples were concentrated using the DNA Clean & Concentrator™-5 Kit (Zymo Research, Irvine, CA, USA), and the concentrations were then determined using the Quant-iT™ High-Sensitivity DNA Assay Kit (Life Technologies). Paired-end sequencing of up to 192 samples were performed on the Illumina MiSeq System (Illumina Inc., CA, USA), including 1.0% PhiX as internal control, using MiSeq Reagent Kits v2 (Illumina Inc., CA, USA). Automated cluster generation and 250 paired-end sequencing with dual-index reads were performed.

Fastq-files demultiplexed by the MiSeq Controller Software were trimmed for amplification primers, diversity spacers and sequencing adapters (biopieces^50^), mate-paired, and quality filtered (USEARCH^51^ v7.0.1090; parameter: -maxee 0.5). UPARSE^52^ was used for OTU clustering (>97% identity) as recommended, in particular removing singletons after de-replication. Chimera checking was performed with USEARCH against the Genomes OnLine Database^53,54^ as recommended. Representative sequences were classified (Mothur^55^ v1.25.0, using the wang function at 0.8 confidence threshold) against the Mothur formatted version of the Ribosomal Database Project^56^ v9. A phylogenetic tree was constructed from an alignment of representative sequences made with Mothur’s align_seqs function against the Greengenes^57^ database (may 2013 version). Alignments were then input to Fasttree^58^ in nucleotide mode.

### Airway immune profiles

Unstimulated airway mucosal lining fluid was obtained from the children at age 1 month by insertion of a pre-casted filter strips based on synthetic absorptive matrix (fibrous hydroxylatedpolyester sheets, Accuwik Ultra (cat no. SPR0730), Pall Life Sciences, Portsmouth, Hampshire, UK) in both nostrils for 2 min, followed by immediate storage at −80 °C. For protein extraction, filter strips from both nostrils were immersed in 300 μl Milliplex assay buffer (cat no. L-AB, Millipore, MA, USA) containing 1 protease inhibitor tablet (Roche) per 25 ml buffer, and then transferred to the cup of a cellulose acetate tube filter (0.22 μm) placed in an Eppendorf tube (Spin X centrifuge tube filter, cat no. CLS8161, Sigma-Aldrich, St Louis, MO, USA). The tube was centrifuged for 5 min at 16,000 × *g*, 4 °C. The obtained protein extract was stored immediately at −80 °C until determination of 20 immune mediators (cytokines and chemokines) using high-sensitivity electrochemiluminescence multiplex assays (Meso Scale Discovery, Rockville, MD, USA)^26,59,60^.

Selection of the immune mediators was decided a priori to represent different types of immune responses: type 1 (Th1/CD8+/natural killer cells/innate lymphoid cell 1 (ILC1)), type 2 (Th2, eosinophils, ILC2), type 17 (Th17, neutrophils, ILC3), and regulatory type responses. Children with excessive nasal secretions on the day of sampling (*n* = 44) were excluded from the analysis.

### Statistical analysis

All data analyses were conducted using the statistical software R v3.3.0^61^. Samples below 2000 reads (*n* = 146, 7.5%) were omitted from the analysis. Sequencing and taxonomy data handling, genus-level agglomeration, alpha diversity (analyzed using Shannon index), and beta diversity (analyzed using Bray–Curtis^62^ and weighted UniFrac^63^ distances) estimates were done using the R-package phyloseq^64^. Samples from children with/without asthma were divided evenly across batch variables (extraction tray, sequencing run). Bootstrap richness estimates were computed as sample-wise median number of OTUs in 100 resamples with replacement at 2000 or 10,000 reads. Differences in alpha diversity were tested using Wilcoxon’s rank-sum tests or linear mixed-effects models when analyzing repeated measures from the same child, using child ID as random intercept, with the effect of time-point calculated as a single-term deletion^65^. Differences in beta diversity were tested using permutational multivariate analysis of variance (PERMANOVA)^66^ with 10,000 permutations.

Differential abundance was tested with Cox proportional hazards regression models using time to asthma diagnosis as outcome, after filtering genera (≥10% presence, ≥0.01% mean relative abundance) and log-transforming relative abundances, using half the lowest nonzero value as a pseudocount. Multiple inference was controlled using the Benjamini–Hochberg FDR approach (*q* values). We used PLS^67^ modeling of asthma by age 6 years as a binary variable using log-transformed, *z*-scaled relative abundances of genera. PLS is a statistical modeling framework specifically well suited for dealing with highly collinear or redundant feature data matrices and excels in situations where the number of features/variables (*p*) are high or even greater than the number of observations (*n*). Similar to, for example, principal component analysis (PCA), features are combined to components via loadings on the original variables, but in a supervised manner to maximize covariance with an outcome, which can be univariate or multivariate. We selected the optimum number of input variables using repeated 10-fold cross-validation of the area under the curve (AUC) statistic to avoid overfitting. The final model was chosen by the highest median AUC value. The predicted values of left out folds were combined to form a bacterial asthma score. Relative importance per taxon was calculated as the median taxon loadings across folds divided by the sum of these median loadings. Associations between the bacterial asthma score and asthma were calculated using Cox proportional hazards regression. The models were not influenced by inclusion of batch variables. Immune mediator concentrations were standardized, total sum normalized per sample, log transformed, and *z*-scored before further analysis. Missing immune mediator values (1.2%) were imputed using the *k*-nearest-neighbors algorithm^68^ prior to normalization. Immune mediators were analyzed in relation to the bacterial asthma score using linear models and cross-validated sparse PLS as described above, adjusted for other bacteria using PCA with four components. Causal mediation analysis was conducted in a model structure with the bacterial asthma score as the predictor, the immune mediator score as mediator and asthma as the outcome^69^. A significance level of 0.05, using two-sided *p* values, was used in all analyses, except PERMANOVA, which is one-sided by design.

## Supplementary information

Supplementary information

Source Data

## Data Availability

The 16S rRNA gene sequencing data is deposited at the Sequence Read Archive (SRA) with the accession no. PRJNA340273. Summary- and feature-level data underlying Figs. 1, 2, and 3 and Supplementary Figs. 1, 3, 4, 6, 7, and 8, and Supplementary Table 3 are provided as a Source Data file. All other data that supports the findings in this study, including clinical data, are available from the corresponding author upon reasonable request: participant-level personally identifiable data are protected under the Danish Data Protection Act and European Regulation 2016/679 of the European Parliament and of the Council (GDPR) that prohibit distribution even in pseudo-anonymized form, but can be made available under a data transfer agreement as a collaboration effort.

## Code availability

The R code used for generating figures and analyses is available from the corresponding author upon request.
