# Supplementary material for: Infant airway microbiota and topical immune perturbations in the origins of childhood asthma
Source: Nat Commun. 2019 Nov 1;10:5001. doi: 10.1038/s41467-019-12989-7 (PMC6825176; doi:10.1038/s41467-019-12989-7)

Supplementary information for the article
Thorsen et al. – "Infant airway microbiota and topical immune perturbations in the origins of childhood asthma"

Contents

Supplementary Table 1: Differential abundances and asthma
Supplementary Table 2: Diversity and differential abundances at 1 week and 3 months
Supplementary Table 3: Contributions of bacterial taxa to the bacterial asthma score
Supplementary Table 4: Associations between inherited and environmental factors and the bacterial asthma score
Supplementary Table 5: Associations between the bacterial asthma score and immune mediators
Supplementary Table 6: Mediation analysis between the bacterial asthma score and immune mediators in their mutual associations with asthma

Supplementary Figure 1: Mean relative genus abundances by sample time point
Supplementary Figure 2: Community structure in the 1-month airway microbiota and asthma
Supplementary Figure 3: Correlations between taxa at one month
Supplementary Figure 4: Cross-validation of sparse Partial Least Squares model
Supplementary Figure 5: Bacterial asthma score composition
Supplementary Figure 6: Immune mediator correlations
Supplementary Figure 7: Adjustment of bacterial asthma score
Supplementary Figure 8: Immune mediator score loadings

Supplementary Table 1: Differential abundances and asthma

Associations between log-relative abundances at one month and asthma development, assessed with Cox proportional hazards regression, as displayed in figure 1. Mean relative abundance and presence (fraction of samples positive for taxon) are given for all taxa, for all samples and stratified by asthma by age six years.

			95% CI				Mean relative abundance			Presence		
**Taxon**	**Phylum**	**HR**	**lower**	**upper**	**P-value**	**q-value**	**All**	**Asthma**	**No asthma**	**All**	**Asthma**	**No asthma**
Veillonella	Firmicutes	1.45	1.21	1.73	0.000041	0.0026	3.1E-02	4.79E-02	2.83E-02	0.98	0.99	0.98
Prevotella	Bacteroidetes	1.32	1.13	1.55	0.00055	0.017	3.7E-03	6.62E-03	2.65E-03	0.67	0.71	0.66
Gemella	Firmicutes	1.28	1.07	1.52	0.0059	0.11	4.1E-02	5.16E-02	3.95E-02	1.00	1.00	1.00
Bacilli	Firmicutes	1.25	1.06	1.48	0.0073	0.11	5.1E-04	7.16E-04	4.66E-04	0.73	0.80	0.72
Bacillales	Firmicutes	1.26	1.06	1.49	0.0085	0.11	1.6E-03	1.77E-03	1.61E-03	0.86	0.88	0.86
Lactobacillus	Firmicutes	1.21	1.03	1.42	0.018	0.19	1.0E-02	1.48E-02	9.23E-03	0.72	0.73	0.72
Streptococcus	Firmicutes	1.22	1.01	1.47	0.034	0.31	3.1E-01	3.32E-01	3.01E-01	1.00	1.00	1.00
Neisseria	Proteobacteria	1.18	1.00	1.38	0.045	0.35	2.3E-02	2.73E-02	2.15E-02	0.89	0.90	0.89
Scardovia	Actinobacteria	1.10	0.96	1.27	0.18	0.85	1.7E-04	2.39E-04	1.72E-04	0.10	0.13	0.10
Campylobacter	Proteobacteria	1.10	0.95	1.28	0.19	0.85	4.6E-04	3.40E-04	5.05E-04	0.24	0.29	0.22
Enhydrobacter	Proteobacteria	0.87	0.71	1.07	0.20	0.85	1.4E-04	1.03E-04	1.69E-04	0.13	0.10	0.14
Lachnospiraceae	Firmicutes	1.11	0.95	1.29	0.21	0.85	9.4E-04	1.35E-03	7.46E-04	0.39	0.46	0.37
Enterococcus	Firmicutes	1.10	0.94	1.29	0.22	0.85	2.1E-03	6.07E-03	9.59E-04	0.32	0.33	0.30
Enterobacteriaceae	Proteobacteria	1.10	0.94	1.30	0.24	0.85	3.2E-02	4.60E-02	2.38E-02	0.88	0.86	0.89
Staphylococcus	Firmicutes	1.10	0.94	1.30	0.24	0.85	2.2E-01	2.07E-01	2.15E-01	1.00	1.00	1.00
Bacteroides	Bacteroidetes	1.09	0.94	1.25	0.27	0.85	5.1E-04	8.95E-05	6.97E-04	0.22	0.27	0.22
Micrococcaceae	Actinobacteria	0.91	0.77	1.08	0.28	0.85	2.8E-03	1.73E-03	2.88E-03	0.59	0.53	0.60
Aquabacterium	Proteobacteria	0.91	0.76	1.09	0.30	0.85	3.2E-04	1.11E-04	4.11E-04	0.23	0.20	0.25
Actinomyces	Actinobacteria	1.08	0.93	1.27	0.32	0.85	1.2E-03	1.55E-03	1.23E-03	0.45	0.46	0.45
Dolosigranulum	Firmicutes	0.92	0.78	1.09	0.32	0.85	1.4E-02	1.36E-02	1.47E-02	0.89	0.87	0.90
Moraxella	Proteobacteria	0.92	0.78	1.09	0.33	0.85	1.3E-01	9.40E-02	1.49E-01	1.00	1.00	1.00
Fusobacterium	Fusobacteria	1.08	0.92	1.27	0.33	0.85	2.3E-03	2.60E-03	2.40E-03	0.46	0.48	0.45
Micrococcus	Actinobacteria	0.92	0.77	1.10	0.34	0.85	1.2E-03	1.17E-04	1.65E-03	0.39	0.37	0.41
Gp6	Other	0.91	0.75	1.11	0.34	0.85	3.4E-04	4.85E-05	4.71E-04	0.15	0.15	0.16
Porphyromonadaceae	Bacteroidetes	1.08	0.92	1.27	0.35	0.85	1.4E-03	1.74E-03	1.37E-03	0.47	0.50	0.47
Tumebacillus	Firmicutes	1.08	0.92	1.26	0.35	0.85	2.9E-04	5.74E-04	1.86E-04	0.22	0.24	0.22
Betaproteobacteria	Proteobacteria	0.91	0.75	1.11	0.37	0.85	1.2E-04	2.70E-05	1.63E-04	0.11	0.10	0.13
Neisseriaceae	Proteobacteria	1.07	0.92	1.25	0.39	0.88	1.9E-04	9.77E-05	2.14E-04	0.19	0.19	0.19
Flavobacteriaceae	Bacteroidetes	1.07	0.91	1.26	0.41	0.88	1.1E-03	1.14E-03	1.15E-03	0.62	0.64	0.62
Pseudomonas	Proteobacteria	1.06	0.91	1.24	0.45	0.88	3.2E-03	2.71E-03	3.64E-03	0.45	0.48	0.45
Staphylococcaceae	Firmicutes	1.06	0.90	1.25	0.46	0.88	2.5E-04	2.59E-04	2.47E-04	0.61	0.63	0.60
Gammaproteobacteria	Proteobacteria	0.94	0.79	1.12	0.48	0.88	1.7E-04	1.41E-04	1.98E-04	0.27	0.25	0.27
Haemophilus	Proteobacteria	1.05	0.90	1.24	0.53	0.88	5.3E-02	3.28E-02	5.76E-02	0.98	0.98	0.98
Atopobium	Actinobacteria	1.05	0.90	1.23	0.53	0.88	4.6E-04	4.83E-04	4.69E-04	0.26	0.25	0.26
Bacteroidetes	Bacteroidetes	1.05	0.89	1.24	0.54	0.88	1.3E-03	1.19E-03	1.21E-03	0.43	0.45	0.43
Rothia	Actinobacteria	1.05	0.89	1.24	0.55	0.88	3.8E-03	4.57E-03	3.88E-03	0.66	0.69	0.66

Chitinophagaceae	Bacteroidetes	0.95	0.80	1.13	0.55	0.88	1.2E-03	1.85E-04	1.66E-03	0.29	0.27	0.30
Acidovorax	Proteobacteria	0.95	0.79	1.14	0.56	0.88	1.9E-04	4.78E-05	2.56E-04	0.11	0.10	0.12
Porphyromonas	Bacteroidetes	0.95	0.79	1.13	0.56	0.88	1.0E-04	3.62E-05	1.00E-04	0.15	0.16	0.14
Gp16	Other	0.95	0.80	1.14	0.58	0.88	2.0E-04	2.23E-05	2.74E-04	0.14	0.14	0.15
Clostridium sensu stricto	Firmicutes	0.95	0.81	1.13	0.59	0.88	5.0E-04	6.54E-05	6.90E-04	0.28	0.31	0.28
Bifidobacterium	Actinobacteria	1.04	0.89	1.22	0.59	0.88	8.7E-04	9.87E-04	9.21E-04	0.33	0.33	0.33
Granulicatella	Firmicutes	1.04	0.89	1.23	0.61	0.88	2.5E-03	2.48E-03	2.62E-03	0.57	0.57	0.57
Rhizobiales	Proteobacteria	0.96	0.80	1.14	0.62	0.88	1.8E-04	1.38E-04	2.06E-04	0.18	0.16	0.19
Leptotrichiaceae	Fusobacteria	1.03	0.88	1.22	0.69	0.94	2.2E-03	3.21E-03	1.89E-03	0.32	0.33	0.32
Myxococcales	Proteobacteria	0.97	0.82	1.14	0.69	0.94	1.5E-04	6.54E-06	2.04E-04	0.14	0.15	0.14
Listeria	Firmicutes	0.97	0.82	1.15	0.75	0.94	2.1E-04	4.07E-05	2.87E-04	0.19	0.21	0.20
Sphingomonas	Proteobacteria	1.02	0.87	1.20	0.77	0.94	9.0E-04	4.95E-04	1.07E-03	0.29	0.32	0.28
Actinomycetales	Actinobacteria	1.02	0.87	1.20	0.77	0.94	3.0E-04	1.59E-04	3.50E-04	0.49	0.50	0.50
Helicobacter	Proteobacteria	0.98	0.82	1.16	0.78	0.94	2.0E-04	1.03E-04	2.54E-04	0.22	0.23	0.22
Acinetobacter	Proteobacteria	0.98	0.83	1.15	0.78	0.94	5.8E-03	4.69E-04	5.97E-03	0.50	0.50	0.50
Arthrobacter	Actinobacteria	0.98	0.82	1.16	0.79	0.94	1.1E-03	8.77E-05	1.58E-03	0.19	0.17	0.19
Streptophyta	Other	1.02	0.87	1.20	0.79	0.94	1.9E-03	2.66E-03	1.79E-03	0.27	0.28	0.27
Rhodobacter	Proteobacteria	0.98	0.83	1.16	0.84	0.98	2.8E-04	9.64E-05	3.66E-04	0.16	0.16	0.17
Bacteria	Other	1.01	0.86	1.19	0.87	0.98	1.3E-03	3.85E-04	1.68E-03	0.50	0.53	0.50
Corynebacterium	Actinobacteria	1.01	0.86	1.19	0.88	0.98	5.9E-02	6.31E-02	5.60E-02	0.99	1.00	0.99
Alphaproteobacteria	Proteobacteria	0.99	0.84	1.17	0.92	0.98	5.2E-04	2.55E-04	6.58E-04	0.29	0.29	0.30
Pelomonas	Proteobacteria	0.99	0.84	1.17	0.93	0.98	5.4E-03	2.31E-03	6.56E-03	0.74	0.79	0.74
Gp3	Other	1.01	0.86	1.18	0.94	0.98	4.0E-04	1.00E-04	5.29E-04	0.21	0.23	0.21
Deinococcus	Other	1.01	0.86	1.18	0.94	0.98	3.8E-04	5.23E-04	3.79E-04	0.17	0.20	0.17
Pasteurellaceae	Proteobacteria	0.99	0.84	1.17	0.95	0.98	1.1E-02	1.13E-02	1.09E-02	0.87	0.87	0.87
Cloacibacterium	Bacteroidetes	1.00	0.85	1.17	0.96	0.98	7.7E-04	1.66E-05	1.09E-03	0.12	0.16	0.11
Ureaplasma	Other	1.00	0.85	1.18	1.00	1.00	1.2E-03	6.03E-05	1.63E-03	0.16	0.17	0.16

Supplementary Table 2: Diversity and differential abundances at 1 week and 3 months

Associations between microbiota diversity, individual microbial taxa and asthma by age 6 years. No results presented here were statistically significant. A) Associations between alpha diversity (measured as boostrap richness estimates at 10,000 and 2,000 reads, and Shannon Diversity index) at 1 week and 3 months and asthma by age 6 years. P-values correspond to Wilcoxon Rank Sum tests. B) Associations between beta diversity (measured as Bray-Curtis or weighted UniFrac distances) at 1 week and 3 months and asthma by age 6 years, tested with Adonis PERMANOVA tests. C) Differential abundance tests between genus log relative abundances at 1 week and 3 months and asthma by age 6 years, tested with Cox proportional hazards regression.

**A**				
**Time**	**Variable**	**statistic**	**P-value**	**n**
1w	richness_10k	17563.5	0.55	497
1w	richness_2k	22030	0.60	497
1w	Shannon	22725	0.99	497
3m	richness_10k	21223.5	0.42	574
3m	richness_2k	28338	0.87	574
3m	Shannon	26508	0.34	574

**B**					
**Time**	**Metric**	**F-statistic**	**P-value**	**R2**	**n**
1w	Bray-Curtis	1.30	0.18	0.0026	497
1w	weighted UniFrac	0.68	0.59	0.0014	497
3m	Bray-Curtis	1.40	0.12	0.0024	574
3m	weighted UniFrac	0.57	0.73	0.0010	574

**C**										
**Timepoint**	**Phylum**	**Genus**	**HR**	**95% CI lower**	**95% CI upper**	**P-value**	**q-value**	**n**	**Mean relative abundance**	**Presence**
1 week	Bacteroidetes	Prevotella	0.74	0.59	0.93	0.01	0.41	544	1.9E-04	0.26
1 week	Fusobacteria	Fusobacterium	0.69	0.51	0.93	0.01	0.41	544	2.4E-04	0.13
1 week	Firmicutes	Veillonella	1.21	1.01	1.44	0.04	0.56	544	1.9E-02	0.92
1 week	Proteobacteria	Pasteurellaceae	1.19	1.01	1.41	0.04	0.56	544	6.1E-03	0.73
1 week	Proteobacteria	Haemophilus	1.19	1.00	1.41	0.05	0.56	544	1.9E-02	0.86
1 week	Proteobacteria	Sphingomonadaceae	0.82	0.66	1.02	0.07	0.63	544	2.5E-04	0.17
1 week	Other	Streptophyta	0.83	0.68	1.03	0.09	0.63	544	2.1E-03	0.22
1 week	Proteobacteria	Escherichia_Shigella	0.81	0.63	1.04	0.10	0.63	544	1.2E-04	0.10
1 week	Bacteroidetes	Cloacibacterium	1.12	0.97	1.29	0.11	0.63	544	8.6E-04	0.10
1 week	Proteobacteria	Neisseria	1.14	0.97	1.35	0.12	0.63	544	1.1E-02	0.79
1 week	Actinobacteria	Micrococcus	0.87	0.72	1.05	0.14	0.63	544	1.3E-03	0.33
1 week	Firmicutes	Bacillales	1.14	0.95	1.37	0.15	0.63	544	2.7E-03	0.89
1 week	Actinobacteria	Actinomyces	0.87	0.72	1.05	0.15	0.63	544	1.6E-03	0.34
1 week	Firmicutes	Veillonellaceae	1.11	0.96	1.29	0.16	0.63	544	7.5E-04	0.18

1 week	Proteobacteria	Sphingomonas	0.88	0.72	1.06	0.18	0.63	544	5.0E-03	0.36
1 week	Firmicutes	Listeria	1.10	0.95	1.29	0.21	0.63	544	1.7E-03	0.27
1 week	Bacteroidetes	Bacteroidetes	0.89	0.73	1.07	0.21	0.63	544	2.9E-04	0.25
1 week	Proteobacteria	Pelomonas	0.90	0.75	1.07	0.22	0.63	544	1.1E-02	0.79
1 week	Firmicutes	Lactobacillus	1.11	0.94	1.31	0.24	0.63	544	8.6E-03	0.62
1 week	Firmicutes	Gemella	1.11	0.93	1.33	0.24	0.63	544	3.4E-02	0.99
1 week	Proteobacteria	Helicobacter	1.10	0.94	1.29	0.24	0.63	544	5.3E-03	0.36
1 week	Firmicutes	Bacilli	1.11	0.93	1.32	0.25	0.63	544	6.8E-04	0.74
1 week	Proteobacteria	Aquabacterium	0.89	0.72	1.09	0.26	0.63	544	4.6E-04	0.12
1 week	Proteobacteria	Methylobacterium	0.89	0.73	1.09	0.26	0.63	544	4.1E-04	0.16
1 week	Proteobacteria	Polyangiaceae	0.89	0.72	1.10	0.27	0.63	544	3.2E-04	0.11
1 week	Firmicutes	Lachnospiraceae	0.90	0.73	1.10	0.29	0.63	544	5.8E-04	0.17
1 week	Proteobacteria	Paracoccus	0.90	0.74	1.10	0.31	0.63	544	8.1E-04	0.17
1 week	Firmicutes	Streptococcus	1.10	0.92	1.31	0.31	0.63	544	1.7E-01	1.00
1 week	Proteobacteria	Acinetobacter	0.91	0.76	1.09	0.31	0.63	544	5.5E-03	0.49
1 week	Proteobacteria	Rhodobacter	1.08	0.92	1.26	0.34	0.64	544	2.1E-03	0.19
1 week	Other	Deinococcus	1.08	0.92	1.26	0.34	0.64	544	3.5E-03	0.24
1 week	Proteobacteria	Comamonas	1.08	0.92	1.25	0.35	0.64	544	7.1E-04	0.15
1 week	Proteobacteria	Moraxella	0.93	0.78	1.11	0.40	0.68	544	8.9E-02	0.99
1 week	Bacteroidetes	Porphyromonadaceae	0.92	0.76	1.11	0.40	0.68	544	7.3E-04	0.28
1 week	Actinobacteria	Micrococcaceae	1.07	0.91	1.27	0.43	0.70	544	2.9E-03	0.55
1 week	Firmicutes	Enterococcus	1.07	0.91	1.25	0.43	0.70	544	2.1E-03	0.28
1 week	Proteobacteria	Alphaproteobacteria	0.93	0.78	1.12	0.45	0.70	544	1.3E-03	0.25
1 week	Proteobacteria	Neisseriaceae	1.06	0.90	1.24	0.48	0.74	544	1.8E-04	0.13
1 week	Actinobacteria	Corynebacterium	0.94	0.79	1.12	0.50	0.74	544	2.6E-02	0.94
1 week	Bacteroidetes	Flavobacteriaceae	0.95	0.79	1.13	0.54	0.79	544	1.0E-03	0.43
1 week	Firmicutes	Dolosigranulum	0.95	0.80	1.13	0.56	0.79	544	1.0E-02	0.76
1 week	Fusobacteria	Leptotrichiaceae	0.95	0.78	1.15	0.58	0.80	544	2.7E-04	0.11
1 week	Proteobacteria	Proteobacteria	1.04	0.89	1.21	0.66	0.86	544	2.9E-04	0.11
1 week	Firmicutes	Tumebacillus	1.03	0.88	1.21	0.69	0.86	544	3.1E-04	0.13
1 week	Firmicutes	Granulicatella	0.96	0.80	1.15	0.69	0.86	544	1.0E-03	0.35
1 week	Other	Bacteria	0.96	0.80	1.16	0.69	0.86	544	3.5E-03	0.32
1 week	Actinobacteria	Bifidobacterium	1.03	0.88	1.22	0.69	0.86	544	1.8E-04	0.15
1 week	Firmicutes	Staphylococcaceae	0.97	0.82	1.15	0.72	0.87	544	9.0E-04	0.82
1 week	Actinobacteria	Rothia	1.03	0.87	1.22	0.74	0.88	544	1.3E-03	0.49
1 week	Actinobacteria	Actinomycetales	0.97	0.82	1.16	0.77	0.89	544	2.7E-04	0.30
1 week	Firmicutes	Salinicoccus	1.02	0.86	1.22	0.83	0.92	544	1.6E-04	0.72
1 week	Bacteroidetes	Bacteroides	1.02	0.86	1.21	0.83	0.92	544	1.1E-03	0.21
1 week	Bacteroidetes	Chitinophagaceae	1.02	0.86	1.21	0.84	0.92	544	2.8E-03	0.24
1 week	Proteobacteria	Gammaproteobacteria	1.01	0.85	1.20	0.92	0.99	544	7.7E-04	0.22

1 week	Proteobacteria	Pseudomonas	1.01	0.85	1.19	0.95	0.99	544	6.9E-03	0.49
1 week	Firmicutes	Clostridium_sensu_stricto	1.00	0.85	1.19	0.97	0.99	544	4.2E-03	0.28
1 week	Firmicutes	Staphylococcus	1.00	0.84	1.19	0.97	0.99	544	4.9E-01	1.00
1 week	Proteobacteria	Enterobacteriaceae	1.00	0.84	1.19	0.99	0.99	544	2.6E-02	0.81
3 months	Proteobacteria	Paracoccus	0.70	0.54	0.92	0.01	0.38	622	3.9E-04	0.19
3 months	Proteobacteria	Escherichia_Shigella	1.18	1.02	1.35	0.02	0.38	622	8.4E-04	0.15
3 months	Proteobacteria	Comamonas	0.78	0.63	0.97	0.02	0.38	622	1.8E-03	0.31
3 months	Proteobacteria	Enterobacteriaceae	1.21	1.02	1.43	0.03	0.38	622	3.1E-02	0.89
3 months	Proteobacteria	Moraxella	0.83	0.70	0.98	0.03	0.38	622	2.4E-01	1.00
3 months	Proteobacteria	Pseudomonas	0.81	0.67	0.99	0.04	0.46	622	3.2E-03	0.40
3 months	Proteobacteria	Neisseria	1.19	1.00	1.41	0.05	0.49	622	3.7E-02	0.95
3 months	Fusobacteria	Leptotrichia	1.14	0.99	1.32	0.07	0.49	622	1.9E-04	0.14
3 months	Proteobacteria	Helicobacter	0.81	0.65	1.02	0.07	0.49	622	5.0E-04	0.19
3 months	Firmicutes	Dolosigranulum	0.86	0.72	1.02	0.08	0.49	622	1.4E-02	0.87
3 months	Bacteroidetes	Chitinophagaceae	0.83	0.67	1.02	0.08	0.49	622	1.4E-03	0.27
3 months	Bacteroidetes	Capnocytophaga	1.13	0.98	1.29	0.09	0.49	622	4.6E-04	0.11
3 months	Proteobacteria	Methylobacterium	0.82	0.66	1.03	0.09	0.49	622	4.4E-04	0.17
3 months	Firmicutes	Staphylococcus	0.86	0.72	1.03	0.10	0.50	622	9.9E-02	0.98
3 months	Bacteroidetes	Bacteroides	0.87	0.72	1.04	0.13	0.62	622	8.2E-04	0.43
3 months	Actinobacteria	Mycobacterium	0.86	0.70	1.06	0.17	0.68	622	3.7E-04	0.17
3 months	Proteobacteria	Neisseriaceae	1.12	0.95	1.32	0.17	0.68	622	1.4E-03	0.40
3 months	Proteobacteria	Aquabacterium	0.88	0.72	1.07	0.20	0.68	622	3.3E-04	0.23
3 months	Proteobacteria	Sphingomonadaceae	0.89	0.75	1.06	0.21	0.68	622	3.5E-03	0.72
3 months	Firmicutes	Megasphaera	0.87	0.71	1.08	0.21	0.68	622	2.3E-04	0.13
3 months	Firmicutes	Anaerococcus	0.87	0.69	1.09	0.22	0.68	622	2.1E-04	0.11
3 months	Firmicutes	Streptococcus	0.90	0.76	1.07	0.23	0.68	622	2.9E-01	1.00
3 months	Bacteroidetes	Prevotella	1.10	0.92	1.31	0.29	0.68	622	1.3E-02	0.88
3 months	Other	Streptophyta	0.90	0.74	1.09	0.29	0.68	622	6.0E-04	0.20
3 months	Proteobacteria	Alphaproteobacteria	0.90	0.74	1.09	0.29	0.68	622	1.0E-03	0.29
3 months	Bacteroidetes	Prevotellaceae	1.09	0.93	1.29	0.30	0.68	622	9.7E-04	0.38
3 months	Actinobacteria	Atopobium	1.09	0.93	1.28	0.30	0.68	622	5.0E-04	0.33
3 months	Proteobacteria	Gammaproteobacteria	0.91	0.76	1.09	0.31	0.68	622	3.9E-04	0.31
3 months	Firmicutes	Lachnospiraceae	0.91	0.76	1.09	0.31	0.68	622	2.7E-03	0.56
3 months	Actinobacteria	Bifidobacterium	0.91	0.76	1.09	0.32	0.68	622	6.5E-04	0.38
3 months	Actinobacteria	Rothia	1.09	0.92	1.29	0.32	0.68	622	1.1E-02	0.81
3 months	Firmicutes	Gemella	0.92	0.77	1.09	0.33	0.68	622	2.2E-02	0.96
3 months	Other	Bacteria	0.91	0.76	1.10	0.34	0.68	622	1.2E-03	0.31
3 months	Proteobacteria	Acinetobacter	0.92	0.77	1.10	0.36	0.68	622	6.2E-03	0.53
3 months	Other	Deinococcus	0.91	0.75	1.11	0.37	0.68	622	1.4E-04	0.13
3 months	Bacteroidetes	Flavobacteriaceae	0.93	0.78	1.10	0.38	0.68	622	1.1E-03	0.59

3 months	Actinobacteria	Corynebacterium	0.93	0.78	1.10	0.38	0.68	622	3.5E-02	0.95
3 months	Firmicutes	Bacillales	0.93	0.78	1.10	0.40	0.68	622	1.4E-03	0.67
3 months	Bacteroidetes	Cloacibacterium	0.92	0.76	1.12	0.42	0.68	622	3.4E-04	0.15
3 months	Bacteroidetes	Porphyromonadaceae	0.93	0.78	1.11	0.43	0.68	622	2.9E-03	0.64
3 months	Actinobacteria	Micrococcus	0.93	0.77	1.12	0.43	0.68	622	5.5E-04	0.33
3 months	Proteobacteria	Sphingomonas	0.93	0.77	1.12	0.43	0.68	622	2.1E-03	0.35
3 months	Firmicutes	Clostridium_sensu_stricto	0.93	0.77	1.12	0.44	0.68	622	1.2E-03	0.30
3 months	Firmicutes	Bacilli	0.94	0.79	1.11	0.45	0.68	622	2.6E-04	0.51
3 months	Firmicutes	Ruminococcaceae	0.93	0.76	1.13	0.46	0.68	622	1.4E-04	0.14
3 months	Other	Ureaplasma	0.93	0.77	1.13	0.46	0.68	622	4.2E-04	0.13
3 months	Proteobacteria	Pasteurellaceae	0.94	0.79	1.11	0.46	0.68	622	1.2E-02	0.89
3 months	Firmicutes	Enterococcus	0.94	0.78	1.13	0.49	0.68	622	5.4E-04	0.31
3 months	Proteobacteria	Polyangiaceae	1.06	0.91	1.23	0.49	0.68	622	4.7E-04	0.10
3 months	Firmicutes	Clostridiales	1.06	0.90	1.25	0.49	0.68	622	2.0E-04	0.27
3 months	Proteobacteria	Haemophilus	1.05	0.89	1.25	0.56	0.76	622	7.0E-02	0.98
3 months	Actinobacteria	Micrococcaceae	1.05	0.89	1.24	0.57	0.77	622	3.1E-03	0.56
3 months	Firmicutes	Granulicatella	0.96	0.80	1.14	0.62	0.80	622	4.0E-03	0.65
3 months	Fusobacteria	Leptotrichiaceae	1.04	0.88	1.23	0.63	0.80	622	4.2E-03	0.50
3 months	Proteobacteria	Rhodobacter	0.96	0.80	1.15	0.63	0.80	622	3.5E-04	0.13
3 months	Proteobacteria	Novosphingobium	0.96	0.80	1.15	0.64	0.80	622	5.1E-04	0.16
3 months	Firmicutes	Lactobacillus	1.03	0.87	1.23	0.71	0.85	622	5.3E-03	0.61
3 months	Actinobacteria	Actinomycetales	0.97	0.81	1.15	0.72	0.85	622	4.7E-04	0.48
3 months	Bacteroidetes	Porphyromonas	0.97	0.81	1.15	0.73	0.85	622	7.3E-04	0.39
3 months	Fusobacteria	Fusobacterium	0.97	0.81	1.15	0.73	0.85	622	1.0E-02	0.81
3 months	Firmicutes	Faecalibacterium	1.03	0.87	1.21	0.76	0.86	622	2.3E-04	0.14
3 months	Proteobacteria	Campylobacter	1.03	0.87	1.22	0.77	0.86	622	8.2E-04	0.35
3 months	Actinobacteria	Actinomyces	0.98	0.82	1.16	0.80	0.89	622	2.4E-03	0.63
3 months	Bacteroidetes	Bacteroidetes	0.99	0.83	1.18	0.89	0.97	622	8.2E-03	0.76
3 months	Firmicutes	Tumebacillus	0.99	0.83	1.17	0.90	0.97	622	2.1E-04	0.15
3 months	Proteobacteria	Pelomonas	0.99	0.84	1.18	0.93	0.99	622	4.9E-03	0.77
3 months	Firmicutes	Listeria	1.00	0.84	1.18	0.96	1.00	622	8.8E-04	0.16
3 months	Firmicutes	Veillonella	1.00	0.84	1.19	0.98	1.00	622	3.2E-02	0.97
3 months	Proteobacteria	Acidovorax	1.00	0.84	1.19	0.99	1.00	622	1.0E-04	0.12
3 months	Proteobacteria	Enhydrobacter	1.00	0.84	1.19	1.00	1.00	622	1.8E-04	0.14

Supplementary Table 3: Contributions of bacterial taxa to the bacterial asthma score

Relative importance is calculated from the median loadings across cross-validation folds in the sparse Partial Least Squares (sPLS) model.

Taxon	Folds selected	Median loading	Relative importance	Mean relative abundance	Presence (fraction positive)
Veillonella	10	0.63	28.1%	0.031	0.98
Prevotella	10	0.53	23.7%	0.0037	0.67
Gemella	10	0.36	16.3%	0.041	1
Bacillales	9	0.27	12.3%	0.0016	0.86
Bacilli	10	0.27	12.2%	0.00051	0.73
Streptococcus	9	0.12	5.4%	0.31	1
Lactobacillus	5	0.046	2.1%	0.01	0.72

Supplementary Table 4: Associations between inherited and environmental factors and the bacterial asthma score

	Estimate	Lower 95% CI	Upper 95% CI	Std. error	P-value	q-value
Sex (Male)	-0.05	-0.22	0.11	0.08	0.54	0.93
Maternal asthma	-0.04	-0.22	0.15	0.10	0.71	0.94
Paternal asthma	0.21	0.00	0.41	0.10	0.047	0.30
Maternal allergy	-0.13	-0.30	0.05	0.09	0.16	0.77
Paternal allergy	0.04	-0.14	0.23	0.09	0.64	0.94
Fish-oil intervention	-0.03	-0.19	0.14	0.08	0.76	0.94
Vitamin D intervention	-0.05	-0.23	0.12	0.09	0.54	0.93
Antibiotics in pregnancy	0.01	-0.16	0.19	0.09	0.88	0.94
Antibiotics at birth (child)	-0.03	-0.53	0.47	0.25	0.90	0.94
Antibiotics at birth (mother)	-0.10	-0.28	0.07	0.09	0.25	0.93
Antibiotic treatment, first month	-0.23	-0.68	0.21	0.23	0.30	0.93
Virus PCR positive, 1 month	0.08	-0.16	0.32	0.12	0.53	0.93
Dog in the home	-0.01	-0.22	0.21	0.11	0.94	0.94
Cat in the home	-0.10	-0.30	0.11	0.11	0.36	0.93
Caesarean section	-0.05	-0.25	0.15	0.10	0.64	0.94
Smoking during pregnancy	-0.10	-0.44	0.23	0.17	0.54	0.94
Season of birth					0.0021	0.04
Winter	0.23	0.01	0.46	0.11	0.041	
Summer	-0.20	-0.45	0.05	0.13	0.11	
Spring	0.03	-0.21	0.27	0.12	0.81	
Breastfeeding at one month	-0.03	-0.23	0.18	0.10	0.80	0.94
Older siblings	-0.17	-0.34	-0.01	0.08	0.042	0.30

Supplementary Table 5: Associations between the bacterial asthma score and immune mediators

Model	Mediator	Type	Estimate	Lower 95% CI	Upper 95% CI	P-value	q-value
Crude	TNF-a	Type 1	0.85	0.78	0.93	0.00033	0.0066
Crude	IL-10	Regulatory	0.87	0.79	0.94	0.00089	0.0077
Crude	IL-1b	Type 17	0.86	0.79	0.94	0.0012	0.0077
Crude	CCL17	Type 2	1.15	1.06	1.25	0.0015	0.0077
Crude	IL-13	Type 2	0.88	0.80	0.96	0.0040	0.016
Crude	CCL2	Type 1	1.14	1.04	1.24	0.0054	0.018
Crude	CCL11	Type 2	1.10	1.01	1.20	0.028	0.079
Crude	IL-2	Regulatory	0.91	0.84	0.99	0.037	0.092
Crude	IL-17A	Type 17	0.92	0.84	1.01	0.075	0.17
Crude	CCL13	Type 2	1.08	0.99	1.18	0.094	0.19
Crude	IL-5	Type 2	1.06	0.97	1.15	0.20	0.34
Crude	CXCL8	Type 17	0.95	0.87	1.03	0.20	0.34
Crude	CCL4	Type 1	0.95	0.87	1.03	0.23	0.35
Crude	TGF-b1	Regulatory	1.04	0.95	1.13	0.42	0.59
Crude	CCL26	Type 2	0.97	0.89	1.06	0.46	0.59
Crude	IFN-g	Type 1	1.03	0.95	1.12	0.48	0.59
Crude	CCL22	Type 2	0.98	0.90	1.08	0.70	0.81
Crude	IL-12p70	Type 1	1.02	0.93	1.11	0.73	0.81
Crude	CXCL10	Type 1	1.00	0.91	1.09	0.96	0.97
Crude	IL-4	Type 2	1.00	0.92	1.09	0.97	0.97
Adjusted	CCL17	Type 2	1.22	1.09	1.37	0.00049	0.0076
Adjusted	IL-1b	Type 17	0.82	0.73	0.92	0.0011	0.0076
Adjusted	CCL2	Type 1	1.22	1.08	1.37	0.0012	0.0076
Adjusted	TNF-a	Type 1	0.83	0.74	0.93	0.0015	0.0076
Adjusted	CCL11	Type 2	1.14	1.02	1.28	0.024	0.097
Adjusted	CCL13	Type 2	1.13	1.01	1.27	0.032	0.11
Adjusted	IL-10	Regulatory	0.90	0.81	1.01	0.072	0.21
Adjusted	IL-13	Type 2	0.90	0.80	1.02	0.093	0.21
Adjusted	CCL22	Type 2	0.90	0.80	1.02	0.096	0.21
Adjusted	IL-12p70	Type 1	1.09	0.97	1.23	0.15	0.30
Adjusted	TGF-b1	Regulatory	1.08	0.96	1.22	0.19	0.33
Adjusted	CCL4	Type 1	0.93	0.83	1.04	0.20	0.33
Adjusted	CXCL10	Type 1	0.94	0.84	1.06	0.32	0.50
Adjusted	IL-17A	Type 17	0.95	0.85	1.07	0.43	0.61
Adjusted	IFN-g	Type 1	1.04	0.93	1.17	0.49	0.64
Adjusted	CCL26	Type 2	0.96	0.86	1.08	0.51	0.64
Adjusted	IL-5	Type 2	0.97	0.87	1.09	0.62	0.72
Adjusted	IL-4	Type 2	1.02	0.91	1.15	0.69	0.76
Adjusted	IL-2	Regulatory	0.99	0.88	1.11	0.88	0.92
Adjusted	CXCL8	Type 17	1.00	0.90	1.12	0.96	0.96

Supplementary Table 6: Mediation analysis between the bacterial asthma score and immune mediators in their mutual associations with asthma

Results from mediation analyses of the shared part of the associations between the bacterial asthma score and the immune mediator score as predictors for asthma. A) Effect estimates are presented for single-predictor model analyses (Individual) and mutually adjusted analyses (Combined). In the overall analyses (Asthma ever by age 6 years), we find only slight attenuation of the estimates for both the bacterial asthma score and the immune mediator score when comparing the individual vs. the combined analyses, thus indicating a low degree of mediation and a large unique contribution to the risk of asthma for both predictors. The same pattern is observed in all the parallel analyses using time-dependent phenotypes (for definitions, please refer to table 2). HR – Hazard ratio (Cox proportional hazards regression), CI – Confidence Interval, OR – Odds Ratio (Logistic regression). Only children with full data available for both the 1 month airway microbiota, the immune mediator score and clinical follow-up were included in these analyses. B) Results from causal mediation analysis, quantifying Average Causal Mediated Effect (ACME) and Average Direct Effect (ADE), and the proportion of mediation (~10%) of the immune mediator score in the effect of the bacterial asthma score on asthma, indicating a significant partial mediation. Results from quasi-Bayesian vs bootstrap estimation were nearly identical. Note that while the name of the analysis contains “causal”, these data are observational.

A

		Bacterial asthma score			Immune mediator score			N
**Asthma phenotype**	**Analysis**	**HR**	**95% CI**	**p-value**	**HR**	**95% CI**	**p-value**	**Cases/Controls**
Overall	Individual	1.42	[1.17-1.71]	0.00032	1.34	[1.11-1.61]	0.0019	117/382
Overall	Combined	1.37	[1.13-1.67]	0.0012	1.29	[1.07-1.55]	0.0077	117/382
		**OR**	**95% CI**	**p-value**	**OR**	**95% CI**	**p-value**	
Transient early	Individual	1.32	[1.02-1.72]	0.035	1.38	[1.07-1.80]	0.013	72/382
Transient early	Combined	1.27	[0.98-1.66]	0.076	1.34	[1.04-1.75]	0.027	72/382
Persistent	Individual	1.69	[1.08-2.72]	0.026	1.38	[0.90-2.13]	0.14	23/382
Persistent	Combined	1.64	[1.04-2.65]	0.039	1.31	[0.84-2.03]	0.23	23/382
Late-onset	Individual	2.20	[1.36-3.71]	0.0019	1.49	[0.96-2.33]	0.075	22/382
Late-onset	Combined	2.13	[1.31-3-62]	0.0032	1.39	[0.88-2.20]	0.16	22/382
Current at 6 years	Individual	1.86	[1.29-2.76]	0.0014	1.48	[1.05-2.11]	0.028	37/382
Current at 6 years	Combined	1.81	[1.24-2.70]	0.0028	1.40	[0.98-2.01]	0.064	37/382

B

Computation method	Effect	Estimate	95% CI	p-value
quasiBayesian	ACME	0.0088	[0.0019-0.0181]	0.0076
quasiBayesian	ADE	0.0726	[0.0286-0.1186]	0.0016
quasiBayesian	Proportion (ACME/Total)	0.1051	[0.0222-0.2824]	0.0078
quasiBayesian	Total	0.0813	[0.0365-0.1287]	0.0002
bootstrap	ACME	0.0088	[0.0020-0.0179]	0.006
bootstrap	ADE	0.0723	[0.0286-0.1223]	0.0012
bootstrap	Proportion (ACME/Total)	0.1087	[0.0232-0.2816]	0.0062
bootstrap	Total	0.0811	[0.0374-0.1322]	0.0002

**Outcome**	Asthma by age 6 years (Yes/No)
**Predictor**	Bacterial asthma score
**Mediator**	Immune mediator score
**Model 1**	Linear regression (Immune mediator score ~ Bacterial asthma score)

**Model 2**	Logistic regression (Asthma6yr(y/n) ~ Immune mediator score + Bacterial asthma score)

Supplementary Figure 1: Mean relative genus abundances by sample time point

Only genera with >1% mean relative abundance at one of the three sample time points are included. Colored by taxonomic phylum, sorted by mean relative abundance at 1 week.

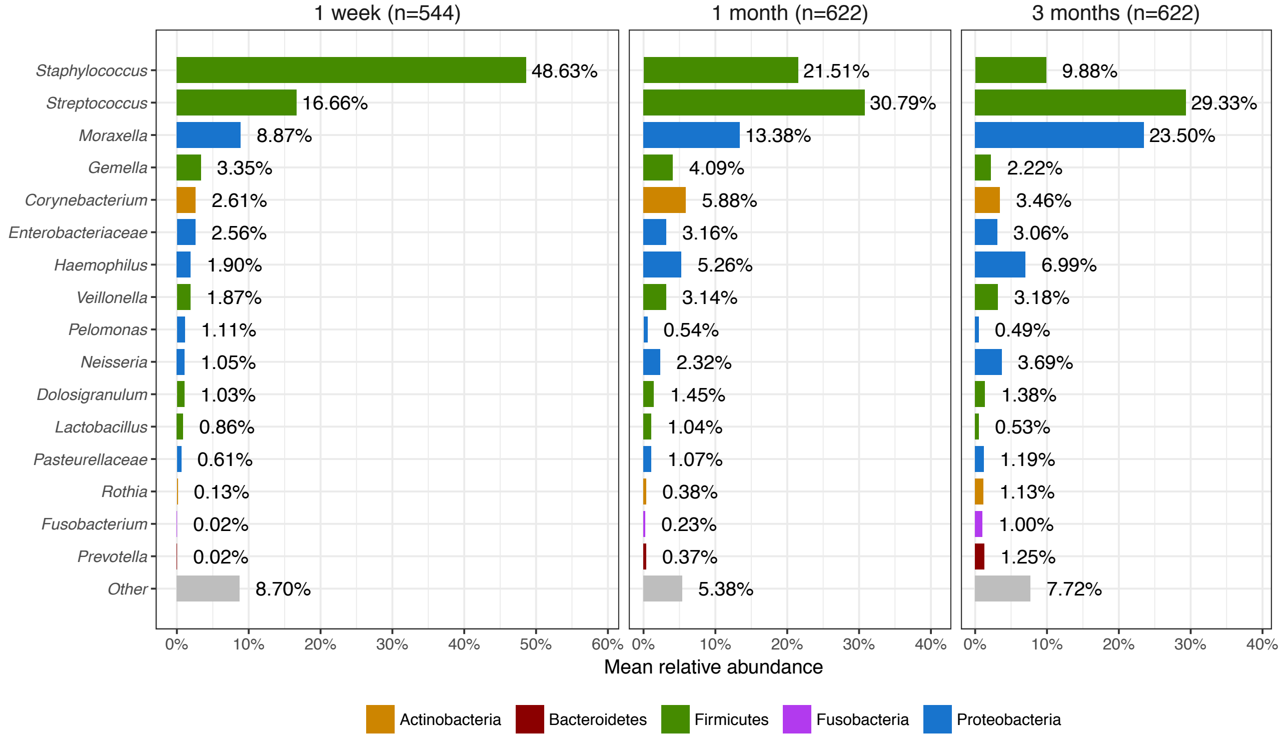

Supplementary Figure 2: Community structure in the 1-month airway microbiota and asthma

Principal Coordinates Analysis (PCoA) plots of Bray-Curtis (A) and weighted UniFrac (B) distances in the 1-month airway microbiota, colored by asthma status by age 6 years. P-values correspond to adonis PERMANOVA tests.

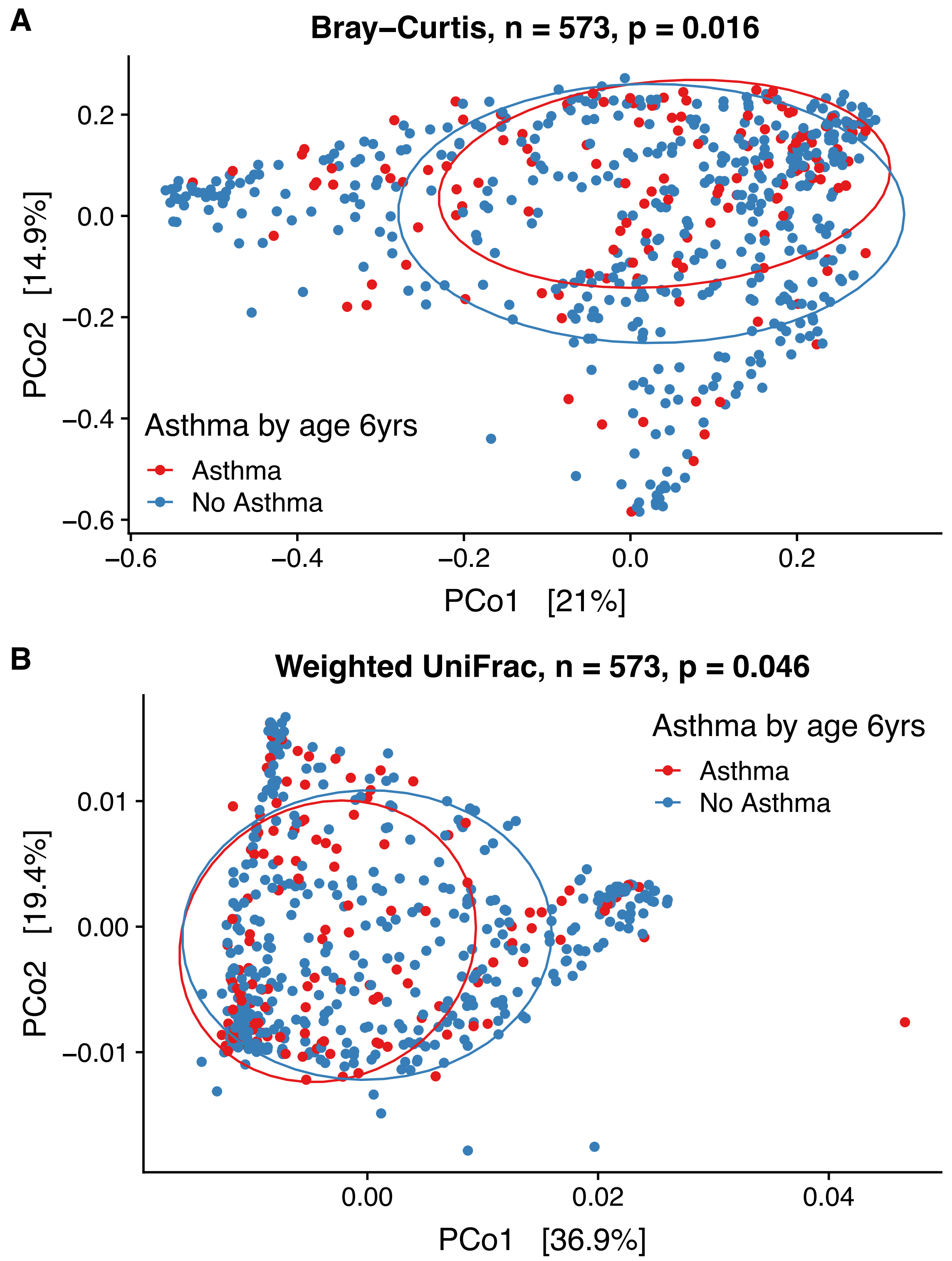

Supplementary Figure 3: Correlations between taxa at one month

Spearman correlation heatmap between genera at one month (n=573), filtered by >10% presence and >10^{-4} mean relative abundance. Values are shown in each cell.

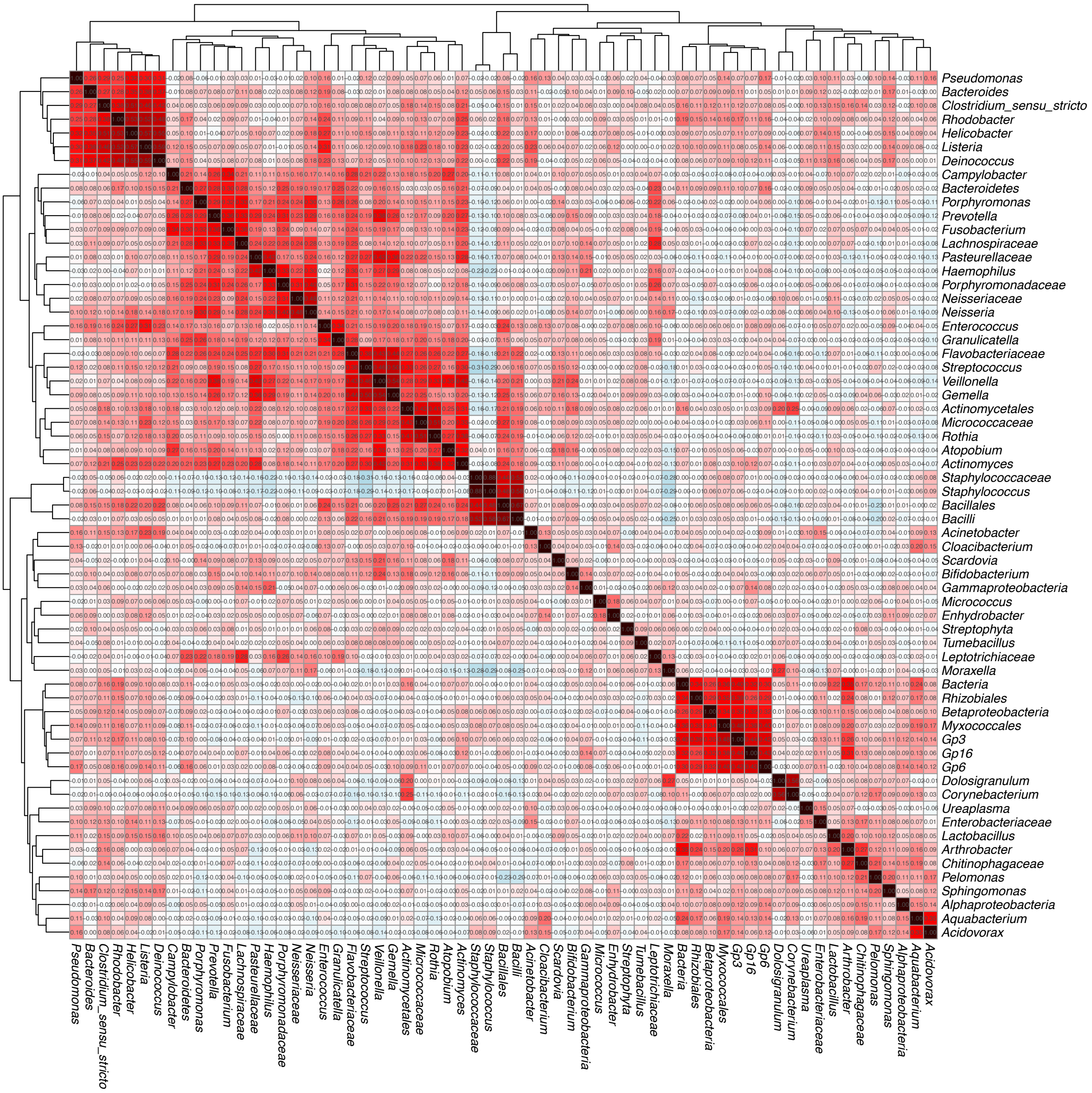

Supplementary Figure 4: Cross-validation of sparse Partial Least Squares model

Cross-validation plot showing AUC results from repeated 10-fold cross-validation of Partial Least Squares models between bacterial genera and asthma, according to number of variables and number of components. Boxplot features: Box denotes 25th and 75th percentile, middle line denotes median, whiskers extend to any data points inside 1.5 x interquartile range from the box. N=573.

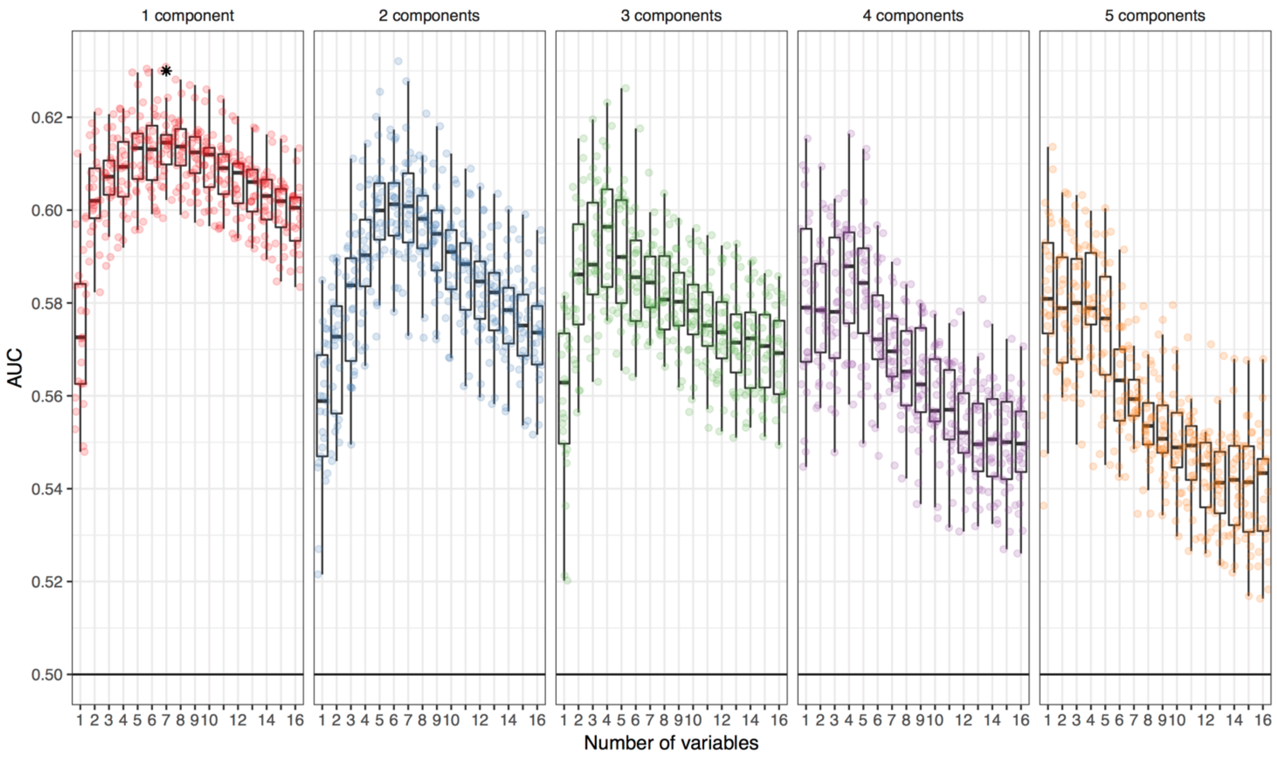

Supplementary Figure 5: Bacterial asthma score composition

Bacterial asthma score and the relative abundances of the seven bacterial genera on which it is based, colored by the tertiles which are used in figure 2. The model is built using repeated cross-validation to avoid overfitting. The relative importance of each taxon is denoted in parentheses. N=573.

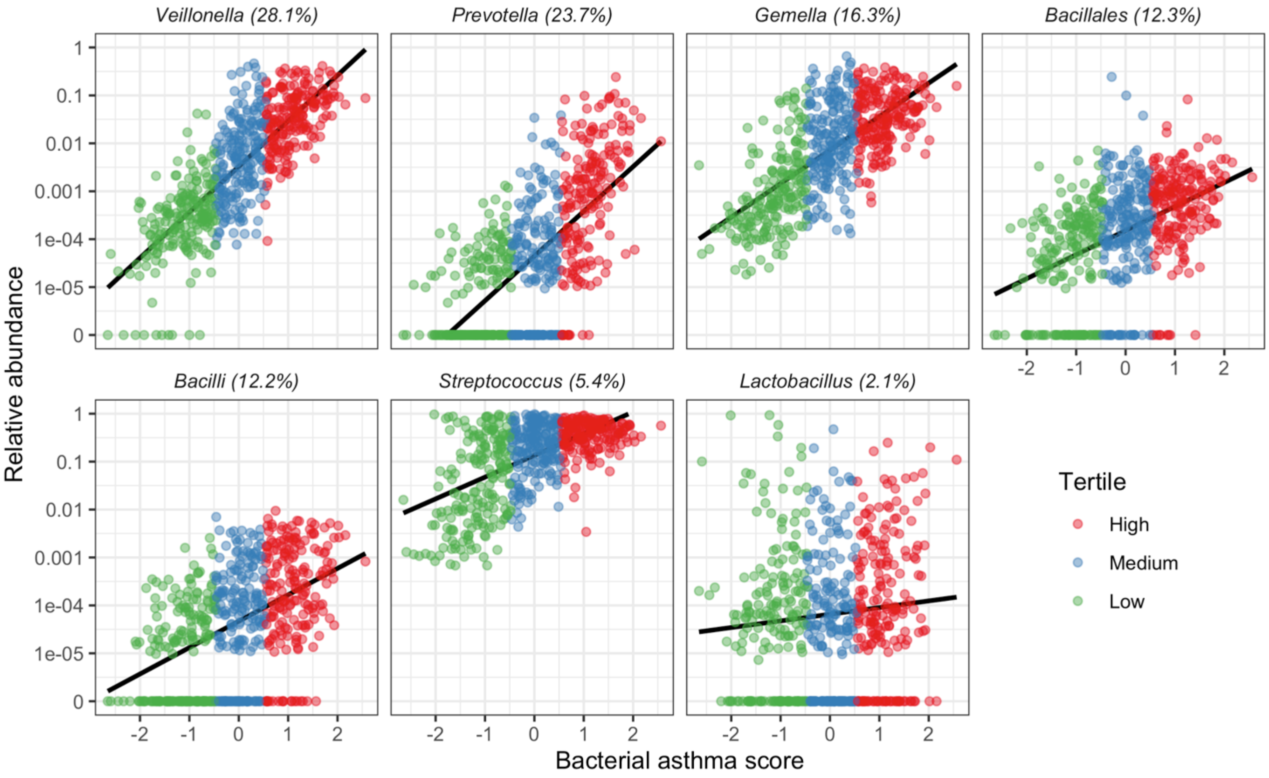

Supplementary Figure 6: Immune mediator correlations

Pearson correlation heatmap between total-sum normalized, log-transformed immune mediator concentrations from nasal mucosal lining fluid at one month of age. Values are shown in each cell. N=499.

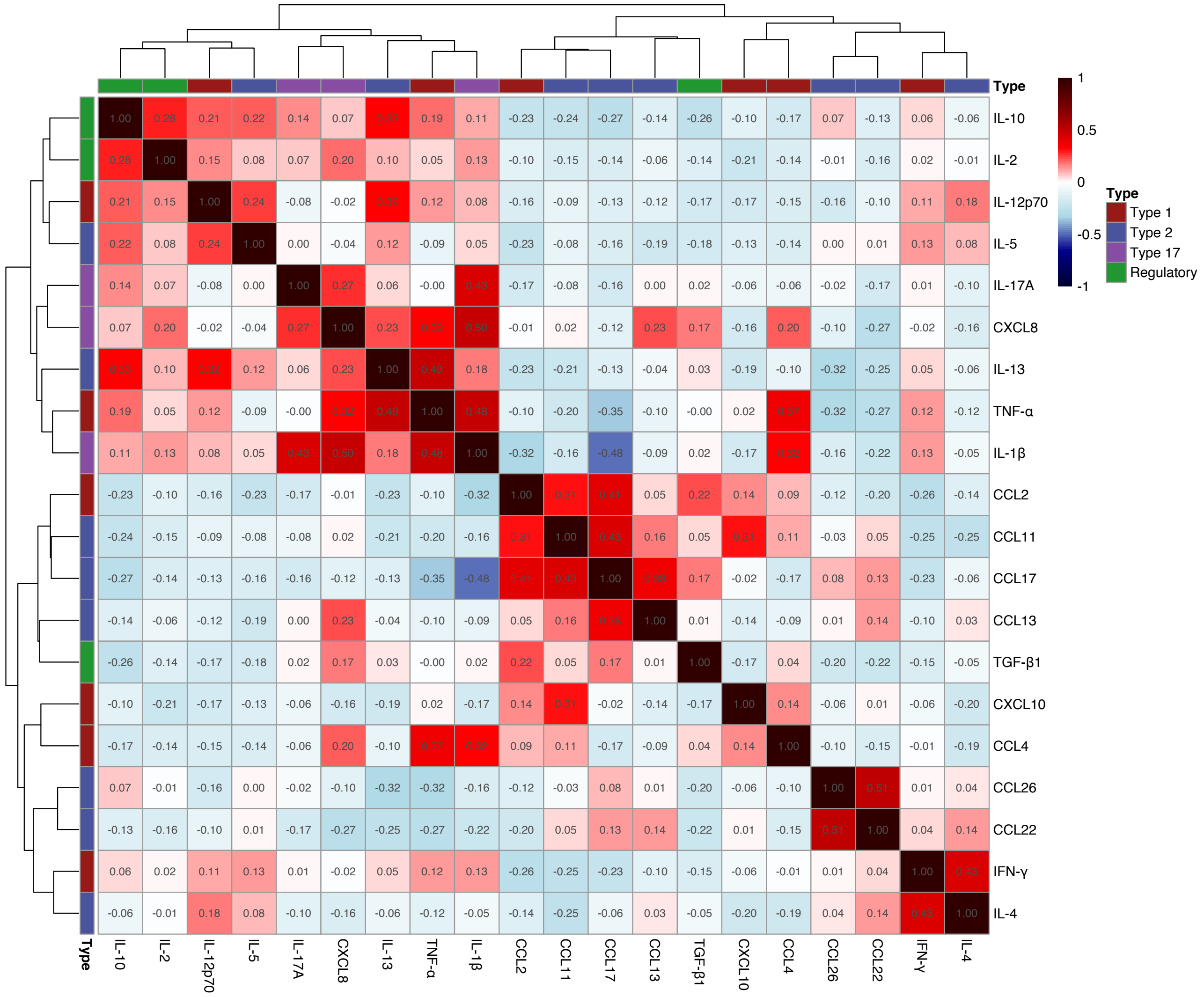

Supplementary Figure 7: Adjustment of bacterial asthma score

Correlation between bacterial asthma score and genus-level relative abundances of all bacteria, before and after successive adjustment for collinearity by Principal Component Analysis (PCA) of all bacteria except *Veillonella, Prevotella, Gemella, Bacilli, Bacillales, Streptococcus,* and *Lactobacillus*. After adjustment for 4 PCs, the majority of the collinearity is removed. Boxplot features: Box denotes 25th and 75th percentile, middle line denotes median, whiskers extend to any data points inside 1.5 x interquartile range from the box. N=499.

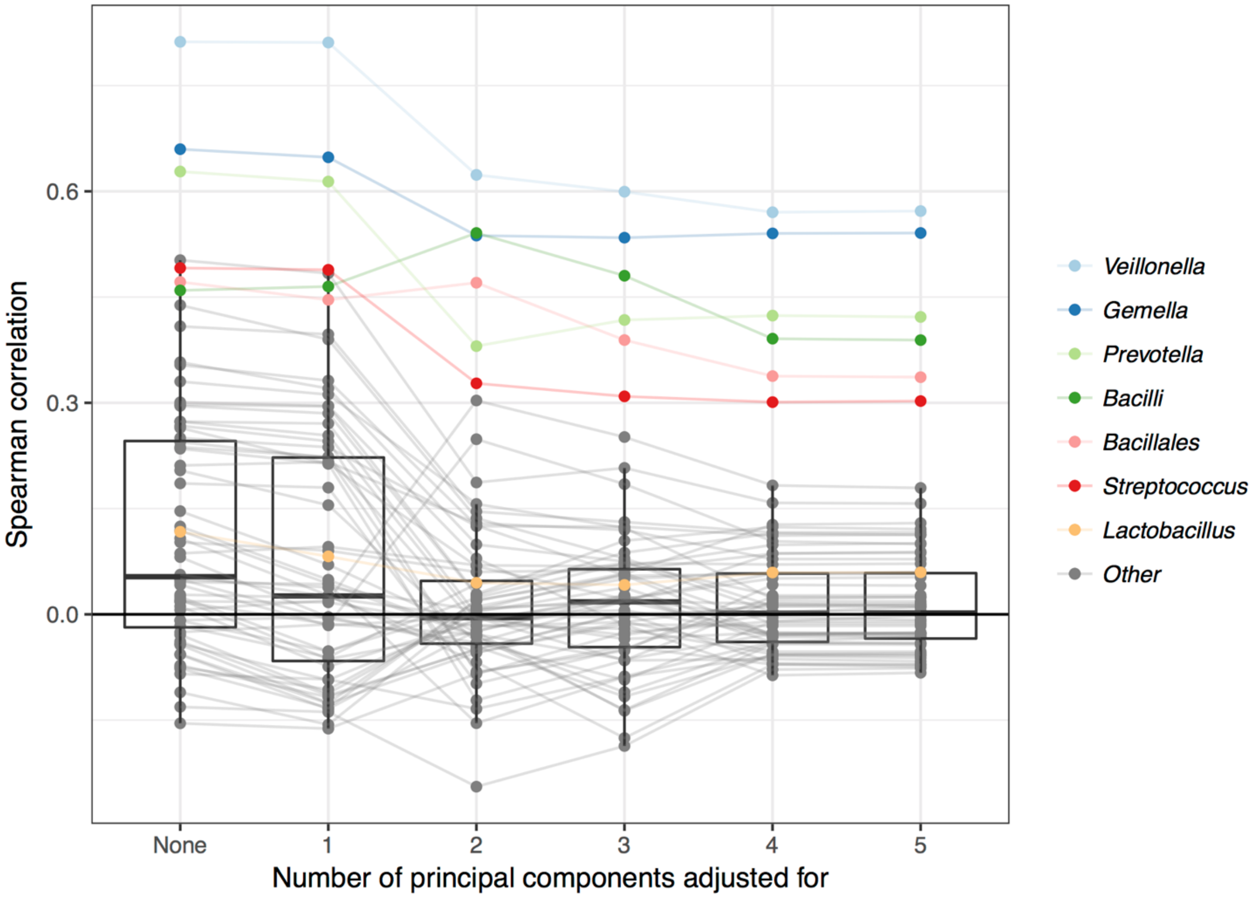

Supplementary Figure 8: Immune mediator score loadings

Loadings from cross-validated sparse Partial Least Squares model between immune mediators and the bacterial asthma score, adjusted for other bacteria. Median values across leave-one-out cross-validation are shown, with standard deviations drawn as error bars. N=499.

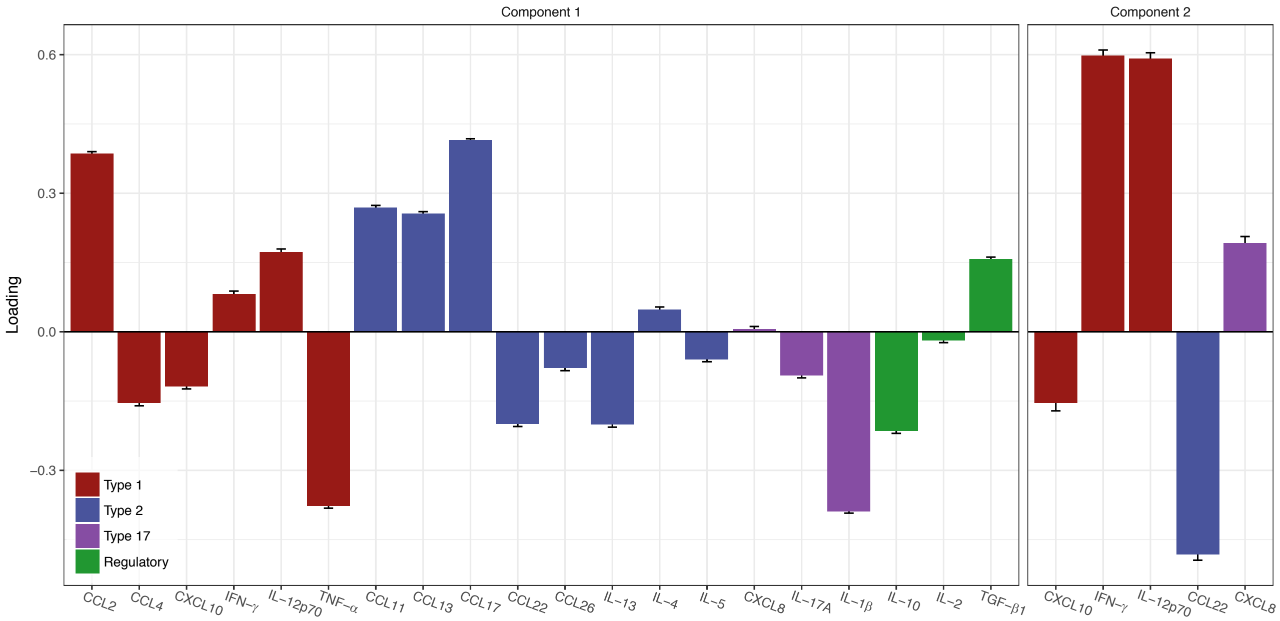

Supplement: Supplementary file 1 — Supplementary information [file 41467_2019_12989_MOESM1_ESM.pdf]
